# Short-Term Grape Consumption Diminishes UV-Induced Skin Erythema

**DOI:** 10.3390/antiox11122372

**Published:** 2022-11-30

**Authors:** John M. Pezzuto, Asim Dave, Eun-Jung Park, Diren Beyoğlu, Jeffrey R. Idle

**Affiliations:** 1 College of Pharmacy and Health Sciences, Western New England University, Springfield, MA 01119, USA; 2 Department of Medicine, UMass Chan Medical School—Baystate, Springfield, MA 01199, USA; 3 Immunology Program, Memorial Sloan Kettering Cancer Center, New York, NY 10065, USA; 4 Division of Pharmaceutical Sciences, Arnold & Marie Schwartz College of Pharmacy and Health Sciences, Long Island University, Brooklyn, NY 11201, USA

**Keywords:** microbiome, metabolomics, skin, bacterial metabolism, DNA damage response, enzyme turnover, radiation biology

## Abstract

Over three million Americans are affected by skin cancer each year, largely as a result of exposure to sunlight. The purpose of this study was to determine the potential of grape consumption to modulate UV-induced skin erythema. With 29 human volunteers, we report that nine demonstrated greater resistance to UV irradiation of the skin after consuming the equivalent of three servings of grapes per day for two weeks. We further explored any potential relationship to the gut–skin axis. Alpha- and beta-diversity of the gut microbiome were not altered, but grape consumption modulated microbiota abundance, enzyme levels, and KEGG pathways. Striking differences in the microbiome and metabolome were discerned when comparing the nine individuals showing greater UV resistance with the 20 non-responders. Notably, three urinary metabolites, 2′-deoxyribonic acid, 3-hydroxyphenyl acetic and *scyllo*-inositol, were depressed in the UV-resistant group. A ROC curve revealed a 71.8% probability that measurement of urinary 2′-deoxyribonic acid identifies a UV skin non-responder. 2′-Deoxyribonic acid is cleaved from the DNA backbone by reactive oxygen species. Three of the nine subjects acquiring UV resistance following grape consumption showed a durable response, and these three demonstrated unique microbiomic and metabolomic profiles. Variable UV skin sensitivity was likely due to glutathione *S*-transferase polymorphisms. We conclude that a segment of the population is capable of demonstrating greater resistance to a dermal response elicited by UV irradiation as a result of grape consumption. It is uncertain if modulation of the gut-skin axis leads to enhanced UV resistance, but there is correlation. More broadly, it is reasonable to expect that these mechanisms relate to other health outcomes anticipated to result from grape consumption.

## 1. Introduction

It is widely accepted that ample consumption of fruits, vegetables, legumes, nuts and whole grains is beneficial for human health. Dietary recommendations vary, but the World Health Organization suggests eating at least 400 g, or five portions, per day [1]. While this may seem simply as “common sense”, there is a solid scientific underpinning in support of such recommendations. Limiting dietary intake of free sugars, saturated fats, and salt, while assuring adequate daily intake of dietary fiber, is known to reduce various human ailments such as metabolic syndrome, cardiovascular disease, and some types of cancer. In addition to the reducing dietary components associated with poor health, the potential to prevent disease and promote good health by chemical constituents within a diet recommended as “healthy” has been extensively studied.

Naturally, any health benefits resulting from dietary habits is a holistic matter, and any type of health benefit realized would be the result of the sum total of materials consumed. Nonetheless, it is of value to study the potential health benefits of individual components based on the assumption of sole, additive or synergistic effects. As a single dietary component, we have elected to focus on the grape. Following our seminal publication regarding resveratrol as a cancer chemopreventive agent [2], numerous studies have following espousing the broad biologic potential of this chemical entity [3]. While the grape is the most common and prevalent source of resveratrol, relatively small quantities result from dietary consumption of grapes or grape products such as wine, whereas the grape itself contains many hundreds of phytochemicals [4]. Since dietary grape consumption is prevalent, as evidenced by the production of over 6 million tons per year in the US alone, it is logical that many potential effects on human health and well-being have been examined. Accordingly, grape consumption has been suggested to have an influence on atherosclerosis, inflammation, cancer, gastrointestinal health, central nervous system (CNS) effects, osteoarthritis, urinary bladder function, and vision [5].

Interestingly, UV absorption by the skin can produce chemical, hormonal, and neural signals, which, in turn, can exert stimulatory effects on the brain and regulate global homeostasis. Based on these modes of action, therapeutic applications of UV radiation have been suggested [6]. Here, however, our main focus is on the potential of human grape consumption to alter skin damage resulting from exposure to UV irradiation. Largely resulting from UV radiation from sunlight, it is estimated that each year over 3 million Americans are affected by nonmelanoma skin cancer (NMSC), including basal cell carcinoma (BCC) and squamous cell carcinoma (SCC). This poses a significant economic burden, but NMSC can be effectively managed in general. Melanoma accounts for about 200,000 cases per year in the US, and about 7600 deaths. Although the genesis of melanoma is multifactorial, the majority of cases are attributed to UV exposure [7]. Again, early diagnosis and treatment is the key to favorable outcome given that the survival rate for patients with malignant metastatic melanoma remains poor [8]. Naturally, primary prevention of skin cancer by blocking or preventing exposure to UV radiation is most effective. Nonetheless, exploration of other ancillary means capable of skin cancer amelioration is worthwhile.

The potential of many phytochemicals to prevent skin cancer have been reported in the literature, with a variety of animal models. This includes constituents of grapes, most notably, resveratrol [2,9]. A substantial number of the studies conducted with laboratory animals involves treatment with chemical carcinogens and tumor promotion, which is highly instructive since important mechanistic insight is provided. It seems reasonable, however, that experiments performed with laboratory animals wherein skin cancer is induced by UV irradiation would be most relevant to the human situation. Further, in regard to grapes, oral administration is viewed of greater relevance than topical application.

An early study suggesting the potential of orally administered grapes to prevent skin cancer induced by UV exposure was provided by Godwin (unpublished, but reviewed with permission in Ref. [4]). Employing UV-irradiated SKH:hr-1 hairless mice, oral administration of a grape powder equivalent to two servings of fresh grapes five times per week significantly reduced edema. After one week, a reduction of about 50% was observed. The reduction increased with time, reaching about 90% after eight weeks. The decrease correlated with photoprotection of lipids in the epidermis. These data provided preliminary, yet intriguing, evidence for the potential of grape consumption to prevent severe skin damage.

More poignant evidence for the potential of orally administered grapes to prevent UV-induced skin cancer, along with mechanistic underpinning, has been provided by elegant studies reported by Ahmad and coworkers. First, with a SKH-1 hairless mouse model using a UVB initiation/promotion protocol, dietary grapes delayed the onset of tumorigenesis, and reduced tumor incidence. These responses were associated with enhanced DNA damage repair, reduced proliferation, increased apoptosis, and modulation of oxidative stress markers [10]. In follow-up studies using tandem mass tag (TMT) quantitative global proteomics, 2629 proteins were modulated by grape feeding, and it was deduced that grape consumption may mitigate tumorigenesis by enhancing protein ubiquitination and degradation caused by oxidative stress [11]. More recently, following short-term exposure to UVB irradiation, grape protected against early-stage epithelial hyperplasia and mast cell infiltration in male and female SKH-1 hairless mice. Long-term studies confirmed that dietary grape reduced tumor counts and malignant conversion, and demonstrated significant decreases in mast cell infiltration, serum IgE and Eotaxin. It was concluded that “Grape powder appears to protect against UVB-mediated skin damage and carcinogenesis in SKH-1 mice and should be explored further as a supplement for NMSC prevention” [12].

Indeed, as an archetypical example of translational research, proof-of-concept has been provided in human clinical studies reported by Elmets and coworkers. Following oral administration of the equivalent of three servings per day of grapes for 2 weeks, the minimal erythema dose (MED) of UV irradiation was increased (i.e., more resistant to sunburn) in 11 volunteers, remained the same in seven volunteers, and decreased in one volunteer [13]. In subsequent studies, significantly lower levels of cyclobutane pyrimidine dimers and double-strand breaks in DNA were observed in volunteers consuming grapes, as well as down-regulation of multiple proinflammatory pathways [14]. These data clearly support the potential of grapes consumed in the diet of humans to provide photoprotection.

Our current objective was to independently confirm the results of Elmets and coworkers in a second clinical investigation. Further, assuming observation of a positive response, an additional objective was to explore unique attributes of responsive human subjects. As described herein, consistent with the results of Elmets and coworkers, consumption of grapes does reduce sensitivity to UV irradiation of the skin in a meaningful portion of the study population. Additionally, relative to the subject population as a whole, the individuals demonstrating greater resistance to UV irradiation could be clearly differentiated based on composite abundance of the microbiota and metabolomic analyses.

## 2. Materials and Methods

### 2.1. Human Subjects, Study Design and Specimen Procurement (Figure 1)

#### 2.1.1. Subject Disposition

From the outset, the goal of this work was to have 30 subjects (15 males, 15 females) complete all phases of the investigation. Forty-one (41) subjects were originally enrolled and 29 subjects completed the study. The reasons for the 12 subjects discontinuing or not completing the study varied (e.g., withdraw of consent, lost to follow-up, health issues unrelated to the study), but were not due to the study protocol. There were no adverse events related to the study protocol or the administration of grape powder. There were no changes to the evaluation of the test site/irradiation site on the back, and subjects were compliant and eligible to continue with study participation.

**Figure 1:**
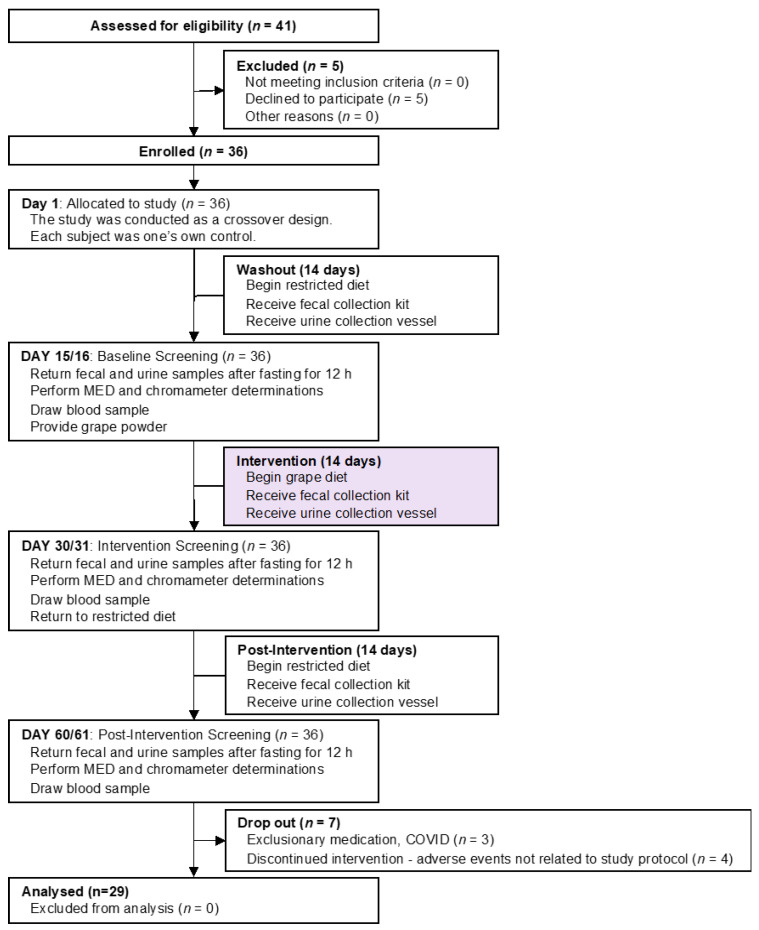
Participant flow chart.

#### 2.1.2. Subject Demographics

Information regarding the 29 subjects completing the study is presented in Table 1. The mean subject age was 41.6 ± 8.2 (SD) years, where the median age was 43.3 years, and the minimum and maximum ages were 24.0 and 55.7, respectively. Thirteen (13) subjects (45%) were female, and 16 subjects (55%) were male. Subject race was White/Caucasian. The majority of subjects, 21 (72%), were not Hispanic or Latino, and the remainder (8) was Hispanic or Latino. The majority of subjects had Fitzpatrick skin type III (25 subjects, 86%), and the remainder was type II (4 subjects, 14%).

#### 2.1.3. Subject Enrollment

First, volunteers for this study were required to meet the inclusion/exclusion criteria described in Supplemental Information (Supplementary File S1). With subjects meeting the criteria, the overall nature of the study was discussed. In particular, the diet and dietary supplement restrictions associated with the study (described in Supplementary File S2) were reviewed, and subjects were given a contact number to ask any questions related to allowable food. At this point, subjects signing an informed consent document were enrolled in the study.

#### 2.1.4. Grape Powder

To assure the consistency and continuity of experimental and clinical research concerning the biological and physiologic potential of grapes, a freeze-dried powder is manufactured under the auspices of the California Table Grape Commission (Fresno, CA, USA) [15]. The grape powder, which serves as a surrogate for fresh grapes, is composed of fresh seeded and seedless red, green and black grapes that are ground and freeze-dried to retain their bioactive compounds. The powder is prepared using good manufacturing procedures for foods. Further quality assurance is provided by assuring the product is contaminant-free through microbial analyses. In addition, the product is subjected to chemical standardization for the quantitation of key phytochemical constituents [15]. For the current studies, vacuum-sealed packets containing 36 g of standardized freeze-dried grape powder were stored at −20 °C until use. Each subject participating in the study received 28 of the packets and were instructed to prepare as directed (Supplementary File S3) and consume two packets per day. Given that the material is simply derived from whole unprocessed grapes, this rate of consumption is equivalent to 3 servings of fresh fruit per day, wherein a single serving is viewed as 126 g (i.e., ¾ cup). All volunteers completing the study protocol were deemed to be compliant with dietary guidelines and grape consumption.

#### 2.1.5. Study Procedure and Specimen Acquisition

Day 1

On Day 1 of the study, diet and dietary supplement restrictions were reviewed with the subjects and they were given a contact number to ask any questions in regard to allowable food. The study diet was started at this time. Subjects were provided with the first fecal sample collection kit. They were instructed to collect a fecal sample on Day 13/14 and return the test kit containing the sample on Day 15 (±2 days). In addition, the subjects were instructed to fast for a 12 h period prior to returning the fecal sample on Day 15, in that plasma would be prepared on this day as well.

On Day 1 of the study, subjects were also provided with a urine collection vessel. On or about Day 14 of the study, each volunteer collected a complete 0–24 h urine sample. During the collection period at home, the sample was refrigerated between and after each collection.

Day 15/16 (referred to as Day 15 in the text)

On Day 15 of the study, the first fecal test kits were received. All fecal samples were collected, recorded, and prepared for shipment according to Diversigen guidelines. Within 24 h, the samples were sent to Diversigen (New Brighton, MN, USA) for analysis by express mail.

In addition, the first urine sample bottles were received and shaken by hand to ensure homogeneity. Volume was measured in mL and recorded. Five mL were transferred to 15 mL Falcon tubes, labelled with the subject’s code, date and time, and 24 h urine volume, and frozen at −20 °C prior to analysis.

UV irradiation was performed, and the following day (Day 16), baseline Minimal Erythema Dose (MED) and chromameter measurements were taken. The sites were photographed as well.

Subjects were provided with the second fecal test kit, a second urine collection vessel, and sufficient grape powder to last the duration of the two-week grape powder consumption time period. In addition to providing instruction sheets (Supplementary File S3), the subjects were verbally instructed on how to use the powder during the study. In brief, just prior to consumption, the subjects mixed the grape powder (36 g) with approximately 6 ounces of water. This was done twice per day (once in the morning and in the afternoon/evening) for 14 days. Subjects were advised if a dose were missed that they should take it as soon as possible. Subjects were given a daily diary in which to record product usage and were instructed to bring in the empty powder pouches to be recorded for compliance on Day 30 (±2 days).

Finally, at least 7 mL of whole blood were drawn into a 10 mL tube heparinized Vacutainer (contained sodium heparin) and placed on ice until centrifugation at 4 °C for at least 15 min at 2200–2500 rpm. Using a glass Pasteur pipette (one time use only), the upper plasma layer (3 mL) was carefully removed without transferring any red cells and placed in a plastic screw-top tube, labeled with subject’s code, date and time. Samples were storied at −20 °C prior to analyses.

Day 30/31 (referred to as Day 30 in the text)

On Day 30 of the study, the subjects returned the second fecal test kit containing a specimen that had been collected in the recent past, and a 24 h urine collection. Samples were processed and stored as described above. Empty pouches that had previously contained the grape powder were returned, and the subjects were interrogated in regard to their experience. No adverse events were reported.

Whole blood was collected and plasma was prepared as described above. Prior to each blood collection, subjects confirmed they had fasted for at least the past 12 h. Subjects were given the third fecal sample collection kit and the third urine collection vessel, and they continued the grape-free study diet and the drug-free regimen for a four-week washout period.

As above, minimal erythema dose (MED) and chromameter measurements and photographs were taken one day after UV exposure.

Day 60/61 (referred to as Day 60 in the text)

On Day 60 (±2 days), subjects returned their final fecal and urine samples, which were prepared for analysis. Final plasma samples were collected, minimal erythema dose (MED) testing was performed, and chromameter measurements and photographs were taken.

#### 2.1.6. Institutional Review Board

The study protocol, the subject information and informed consent form (ICF), and other written subject information were reviewed and approved by the Institutional Review Board (IRB) IntegReview, 3815 S. Capital of Texas Hwy, Suite 320, Austin, TX 78704, USA, Phone: 512-326-3001, Fax: 512-697-0085.

All research was performed in accordance with relevant guidelines and regulations; informed consent was obtained from all participants.

### 2.2. Dermatologic Treatments and Evaluations

#### 2.2.1. Light Source

The light source was a Xenon Arc Solar Simulator with an UV reflecting dichroic mirror, UVC blocking filter, and visible/infrared blocking filter that generated a continuous emission spectrum in the UVA and UVB range (290 to 400 nm). The output was measured daily prior to irradiation using a radiometer/photometer.

#### 2.2.2. Minimal Erythema Dose (MED) Testing

Exposure to UVB radiation causes a small area of skin to be sunburned (approximately 1 cm in diameter). In order to determine the MED, the reaction of the skin was recorded 24 h after exposure. The minimal dose that induced uniform visible reddening up to the borders of the exposure site was defined as the MED. The MED irradiation/24 h evaluation was repeated if needed with either a higher or lower UV dose in order to capture the MED response. Subjects were scheduled (±2 days) for a repeat MED visit. After determining the MED for an individual at baseline (Days 15/16), he/she was asked to ingest grape powder for two weeks. At that time, the MED testing was again performed to determine if the amount of ultraviolet light required for the MED had changed. Following a washout diet, MED was determined again.

On Day 15, subjects had an area of skin on their back, approximately 50 cm^2^, divided into 6 equal sites marked with a surgical marker. The duration of UVA/UVB irradiation for MED exposure was calculated based on subjects’ Fitzpatrick Skin Type and the output of the solar simulator. The solar simulator output was measured prior to each irradiation. Details of the UV irradiation including output of the simulator, time of exposure, equipment used, and staff performing irradiations were documented.

Six 1 cm areas of skin on the mid to lower back were exposed gradually to increasing doses of ultraviolet light (290–400 nm) using a full spectrum UV light (Solar Light Company multiport solar simulator, Glenside, PA, USA). Each of the site’s exposure differed from the next. The doses of UV aligned with the studies reported by Elmets [13,14] using a similar dose range for exposure of the MED. The sites were administered a range of UV exposure with each successive dose increasing as follows: 2.0×, 1.5×, 1.33×, 1.25×, and 1.20×. The doses administered for the MED testing were based on standardized protocols used for subjects participating in clinical research studies using a Xenon Arc Solar Simulator and varied depending on the Fitzpatrick sun reactive skin type. Approximately 24 h later, the area of skin in which there was uniform erythema was considered the MED.

#### 2.2.3. Dermatologic Evaluations

At the Baseline (Day 15) and Day 30 and Day 60 evaluations, subjects underwent minimal erythema dose (MED) irradiation testing on their mid to lower backs to determine their sensitivity to UV light and the sunburn response. Twenty-four hours after the UV exposure, subjects returned for evaluation of the test sites to determine the MED by a trained dermatologic evaluator using the following grading scale: 0, no visible reaction; 0.5, slight, patchy erythema; 1, minimally perceptible skin reddening, uniform redness up to the borders of the exposure site (pink) (MED); 2, moderate erythema (definite redness); and 3, strong erythema (very intense redness).

#### 2.2.4. Definition of MED

Minimal erythemal dose was defined as the smallest UV dose that produced perceptible redness of the skin (erythema) with clearly defined borders at 16–24 h after irradiation using a standardized filtered UV light source that emitted UVA/UVB (full spectrum 290 to 400 nanometers) irradiation as part of its emission spectrum.

#### 2.2.5. Chromameter Measurements

To quantitatively assess erythema and pigmentation, colorimetry was performed with a chromameter (Konica Minolta Chromameter, CR-400, Ramsey, NJ, USA). The chromameter measurements (L*, a*, b*) were taken at Day 16 at the site selected for subsequent MED determinations. This was repeated at Day 32, and Day 61, at the MED site. Three readings were taken and averaged at each time point. The L* value is for dark to light. The lighter the skin, the higher the value. Lower values can indicate tanning. The a* (redness) values: increased value/higher redness, lower values/higher green. The b* (yellowness) values: increased value/higher yellow, decreased value/blue.

### 2.3. Treatment of Fecal Microbiota and Microbiome Analysis

#### 2.3.1. DNA Extraction

Fecal samples were analyzed by Diversigen (New Brighton, MN, USA) [16]. Samples were extracted with PowerSoil Pro (Qiagen, Hilden, Germany) automated for high throughput on the QiaCube HT (Qiagen), using Powerbead Pro Plates (Qiagen) with 0.5 mm and 0.1 mm ceramic beads.

#### 2.3.2. DNA Quantification QC

Samples were quantified with Quant-iT PicoGreen dsDNA Assay (Invitrogen, Waltham, MA, USA).

#### 2.3.3. Library Preparation and Sequencing

Libraries were prepared with a procedure adapted from the Illumina DNA Prep kit (Illumina, San Diego, CA, USA). For BoosterShot^®^ (Shallow Sequencing, 2 M reads/sample), libraries were sequenced on an Illumina NovaSeq using single-end 1 × 100 reads (Illumina).

#### 2.3.4. Sequence Quality Control

DNA sequences were filtered for low quality (Q-Score < 30) and length (<50), and adapter sequences were trimmed using Cutadapt. Human host sequence reads were removed using Bowtie2.

#### 2.3.5. Taxonomic Annotation

For the assessment of taxonomic annotation, sequences were trimmed to a maximum length of 100 bp prior to alignment and converted to a single fasta using shi7. DNA sequences were aligned to a curated database containing representative genomes in RefSeq for bacteria with additional manually curated strains (Venti). Alignments were made at 97% identity against all reference genomes. Every input sequence was compared to every reference sequence in Diversigen’s Venti database using fully gapped alignment with BURST. Ties were broken by minimizing the overall number of unique Operational Taxonomic Units (OTUs). For taxonomy assignment, each input sequence was assigned the lowest common ancestor that was consistent across at least 80% of all reference sequences tied for best hit. Samples with fewer than 10,000 sequences were also discarded. OTUs accounting for less than one millionth of all species-level markers and those with less than 0.01% of their unique genome regions covered (and <1% of the whole genome) were discarded. The number of counts for each OTU was normalized to the average genome length. Count data were then converted to relative abundance for each sample. The normalized and filtered tables were used for all downstream analyses.

#### 2.3.6. Functional Annotation

Kyoto Encyclopedia of Genes and Genomes Orthology groups (KEGG KOs) were observed directly using alignment at 97% identity against a gene database derived from the Venti strain database. To construct this database, a representative strain for each species in the Venti database was annotated using Prokka (v 1.12). Prokka annotations were cross referenced to KEGG IDs, and gene sequences with KEGG annotations were retained for use in the functional database. The KO table and downstream tables contain the directly observed KO counts converted to relative abundance within a sample. KOs were collapsed to level-2 and -3 KEGG pathways and KEGG Modules.

#### 2.3.7. Alpha- and Beta-Diversity

The Chao1 index, Shannon Index and observed OTU count (taxonomic group) were calculated using a rarefied, filtered taxonomy table set to the minimum depth allowed for a sample (10,000) using QIIME 1.9.1. Bray–Curtis beta diversity metrics were calculated from the filtered taxonomy and KEGG module/enzyme relative abundance using QIIME 1.9.1.2.

### 2.4. Urine and Plasma Metabolomics

#### 2.4.1. Plasma GC-MS Metabolomics

As described previously [17,18], quality control (QC) samples were prepared by pooling 50 μL aliquots of all the plasma samples, and every sample was divided into 200 μL aliquots and stored in Eppendorf tubes. All samples were analyzed in duplicate and together with the QC samples. Samples were derivatized with BSTFA/TMCS (Millipore Sigma, Burlington, MA, USA) and analyzed with an Agilent GC-MS (Agilent, Santa Clara, CA, USA). Chromatographic peaks were identified using AutoQuant (Agilent, Santa Clara, CA, USA) that compared their mass spectra with the NIST 14 spectral library of 242,466 mass spectra. In cases of ambiguity where related metabolites produced similar mass spectra, authentic standards were employed and the peaks identified from their retention times on the gas chromatographic column. Some components produced more than one derivative (and therefore chromatographic peak), which were summed. The relative concentration of each metabolite was determined from the ratio of its peak area to the peak area of the internal standard 4-chlorophenylacetic acid. An Excel spreadsheet was then constructed using Quant Browser (Leoson BV, Middelburg, The Netherlands) that contained the peak area ratio (relative concentration) of all identified metabolites in all samples. This data matrix was imported into SIMCA 17 (Sartorius Stedim Data Analytics AB, Umeå, Sweden) in order to conduct multivariate data analysis.

#### 2.4.2. Urine GC-MS Metabolomics

First, because of its well established predominance in GC-MS chromatograms of derivatized urine, urea was removed from all urine samples by incubation with Jack bean urease. This procedure had been reported to increase the number of metabolites detected in urine and reduce their coefficient of variation [19]. In all other respects, the urine samples were analyzed over seven consecutive days by the same procedures as the plasma samples.

#### 2.4.3. Orthogonal PLS-DA Analysis (OPLS-DA)

This analysis reduces the dimensionality of the PLS-DA scores plots. Consequent OPLS-DA loadings S-plots show the metabolites determined by GC-MS in relation to their relative abundance (X-axis) and their correlation to the OPLS-DA model (Y-axis). Loadings in the upper right quadrant represent metabolites that are upregulated in the test group and those in the lower left quadrant represent metabolites that are downregulated in the test group. Loadings that straddle the graphical point (0,0) represent metabolites that are unrelated to the experimental manipulation, e.g., change of diet.

### 2.5. Sub-Analyses of UV-Resistant Study Participants

After performing MED studies, it was discovered that 9 of the 29 participants demonstrated increased resistance to UV irradiation at Day 30 (following grape consumption), whereas the remaining 20 participants were not responsive. Further, 3 of the 9 UV-resistant participants continued to demonstrate resistance at Day 60, whereas the remaining 6 participants who demonstrated resistance at Day 30 did not demonstrate resistance at Day 60. Sub-analyses were performed comparing these 9 UV-resistant subjects with the 20 remaining subjects, as well as the 3 subjects retaining UV-resistance at Day 60 with the 6 remaining subjects, as well as the 20 non-responders, using the same methodology as described above.

### 2.6. Statistical Analyses

Based on the results of Elmets et al. [13,14], a dichotomous endpoint was projected for this one-sample study. Elmets et al. reported a response rate of 57%; we projected a response rate of 25%. Setting alpha and beta at 0.05 (95% power) would require a sample size of 28. To determine the statistical significance between the groups over time, paired and unpaired *t*-tests or Mann–Whitney *U*-tests were performed. In addition, Cohen *d*-values (effect size) were computed to compare different groups. Univariate data analysis (Wilcoxon matched-pairs signed rank test) was performed using GraphPad Prism 9.3.1 (GraphPad Software, San Diego, CA, USA) for analyzing GC-MS metabolomics. Multiple *p*-values were analyzed using the Benjamini–Hochberg procedure to control for false discover rate (FDR). The resulting values, designated *Q*, were considered as statistically significant when *q* < 0.05. However, for general interest, in some cases, *q* values exceeding 0.05 are reported, along with Cohen’s *d* effect size [20] when possible. Additional methods of statistical analyses are included in the text.

## 3. Results

### 3.1. MED Testing

The entire study protocol was completed by 29 subjects (Table 1). Considering the group as a whole, minimal erythema dose (MED) irradiation testing yielded a baseline of 40.25 ± 11.27 (±SD) mJ, which was increased to 43.11 ± 11.37 mJ following grape consumption on Day 30 (two-tailed *p* = 0.0018). Following a one-month washout period, on Day 60, the MED was 41.05 ± 11.89 mJ (*p* = 0.49 relative to baseline, paired *t*-test), indicating the protective effect was not durable.

Notably, however, of the 29 subjects, at Day 30, a total of nine subjects (31%) demonstrated an increase in the MED relative to the baseline, whereas the remaining 20 subjects demonstrated no change whatsoever (Δ0) (Table 1). From the outset of the protocol (Day 15), the MED of these nine responsive subjects was not different from the MED of the entire group (35.86 ± 7.94 vs. 40.11 ± 11.27 mJ, *p* = 0.30, unpaired *t*-test; *p* = 0.20 Mann–Whitney *U*-test). Within the group of nine responders, however, the baseline MED was significantly increased on Day 30 (following grape consumption) from 35.86 ± 7.94 to 44.81 ± 9.92 mJ (*p* < 0.0001). On Day 60, three of the nine responders retained MED resistance of the same magnitude as observed on Day 30, whereas the remaining six returned to the same sensitivity as observed in the baseline test on Day 15 (Table 1). Among the 20 others in the subject population, at Day 60, 18 continued to show no change from baseline (Δ0), and two actually showed increased sensitivity (31.70 mJ reduced to 25.40 mJ, and 49.60 mJ reduced to 39.70 mJ).

### 3.2. Chromameter Testing

Chromameter measurements were taken at the MED site based on the Commission Internationale de l’Eclairage (CIE) L*a*b* color scale [21]. Evaluating the subject group as a whole (*n* = 29), chromatic color a* (green to red axis) was 10.98 ± 2.07 on Day 15. This value was not significantly changed on Day 30 (10.69 ± 1.94; *p* = 0.36; paired *t*-test) or Day 60 (10.37 ± 2.20; *p* = 0.12). Similarly, the chromatic color b* (blue to yellow axis) was 15.86 ± 3.46 on Day 15, and not significantly changed on Day 30 (15.73 ± 3.23; *p* = 0.66) or Day 60 (15.69 ± 3.5; *p* = 0.59). The achromatic color L* (black to white axis) was 63.33 ± 2.46 on Day 15, unchanged on Day 30 (63.84 ± 2.67; *p* = 0.45), but somewhat elevated on Day 60 (64.71 ± 2.39; *p* = 0.028).

A comparison of the nine responders to the remaining 20 subjects was then performed. For a*, the values were as follows: Day 15, 10.90 ± 0.50 (*n* = 9) vs. 11.02 ± 2.32 (*n* = 20) (*p* = 0.63; Mann–Whitney *U*-test); Day 30, 10.26 ± 1.82 (*n* = 9) vs. 10.88 ± 2.01 (*n* = 20) (*p* = 0.67); Day 60, 9.83 ± 1.57 (*n* = 9) vs. 10.61 ± 2.19 (*n* = 20) (*p* = 0.42). For b*, the values were as follows: Day 15, 15.95 ± 3.43 (*n* = 9) vs. 15.77 ± 3.52 (*n* = 20) (*p* = 0.98); Day 30, 15.61 ± 3.91 (*n* = 9) vs. 15.79 ± 2.99 (*n* = 20) (*p* = 1.0); Day 60, 15.82 ± 4.20 (*n* = 9) vs. 15.64 ± 3.28 (*n* = 20) (*p* = 0.81). For L*, the values were as follows: Day 15, 63.40 ± 2.39 (*n* = 9) vs. 63.45 ± 2.55 (*n* = 20) (*p* = 0.98); Day 30, 63.75 ± 2.40 (*n* = 9) vs. 63.48 ± 4.03 (*n* = 20) (*p* = 0.73); Day 60, 64.91 ± 1.76 (*n* = 9) vs. 64.71 ± 2.66 (*n* = 20) (*p* = 0.73).

To explore the colorimetric changes a bit deeper with the subject group as a whole (*n* = 29), the extent of average changes (Δ) in the L*a*b* color scale were evaluated at Day 30 and Day 60 relative to baseline. Chromatic color Δa* at Day 30 was −0.291 ± 1.69 and at Day 60 was −0.470 ± 1.95 (*p* = 0.71; paired *t*-test). The corresponding values for the chromatic color Δb* at Day 30 was −0.090 ± 1.48 and at Day 60 was −0.130 ± 1.62 (*p* = 0.91). Finally, the achromatic color ΔL* yielded +0.408 ± 1.43 on Day 30 and +1.34 ± 1.69 on Day 60 (*p* = 0.027).

The same general exercise was performed with the nine subjects who demonstrated significantly enhanced MED values. On Day 30 and Day 60, the respective Δa* values of the nine responders were −0.638 ± 1.73 and −1.067 ± 1.93 (*p* = 0.51; Mann–Whitney *U*-test). The Δa* values for the remaining 20 subjects on Day 30 (−0.140 ± 1.68) or Day 60 (−0.401 ± 2.06) did not differ from the nine responders (*p* = 0.32 and *p* = 0.23 on Day 30 and 60, respectively). On Day 30 and Day 60, the respective Δb* values of the nine responders were −0.341 ± 1.30 and −0.132 ± 1.92 (*p* = 0.93). The Δb* values for the remaining 20 subjects on Day 30 (0.0225 ± 1.58) or Day 60 (−0.129 ± 1.53) did not differ from the nine responders (*p* = 0.98 and *p* = 0.73 on Day 30 and 60, respectively). On Day 30 and Day 60, the respective ΔL* values of the nine responders were 0.366 ± 1.29 and 1.51 ± 1.57 (*p* = 0.042). The ΔL* values for the remaining 20 subjects on Day 30 (0.428 ± 1.52) or Day 60 (1.26 ± 1.77) did not differ from the nine responders (*p* = 0.87 and *p* = 0.91 on Day 30 and 60, respectively).

Thus, in total, the results of the color analysis revealed no significant changes during the course of the study other than a slight elevation of the L* value at Day 60. Further, color analysis at each time point performed with the nine subjects found to be capable of greater resistance to UV irradiation showed no significant differences relative to the remainder of the study population.

### 3.3. Microbiome Analyses

As described previously, the alpha- and beta-diversity of the microbiome with these 29 volunteers who completed the study protocol was assessed and no significant differences were found when comparing Days 15, 30 and 60 [16]. In the current study, we investigated the alpha- and beta-diversity of the nine subjects showing resistance to UV irradiation. As is the case with the group as a whole, no significant differences were observed in alpha-diversity based on OTUs, Chao1 or Shannon analyses on the three respective days (Supplementary File S4, Figure S1). Similarly, no significant differences were observed in beta-diversity based on cluster analysis of PCA or PCoA plots (Supplementary File S4, Figure S2).

**Figure 2:**
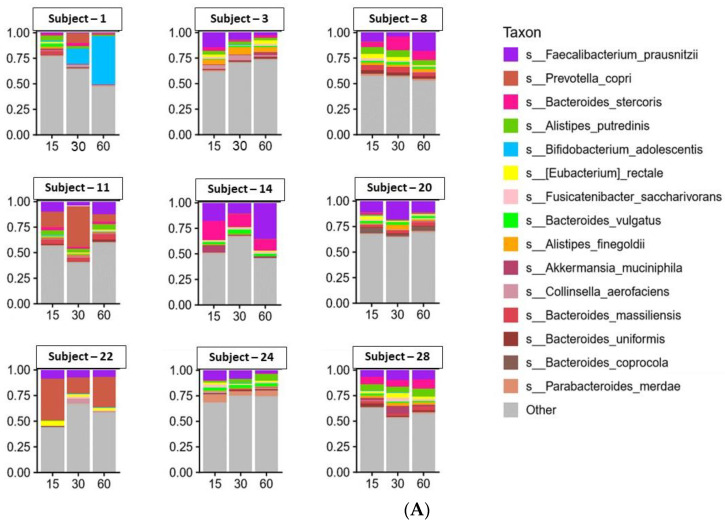

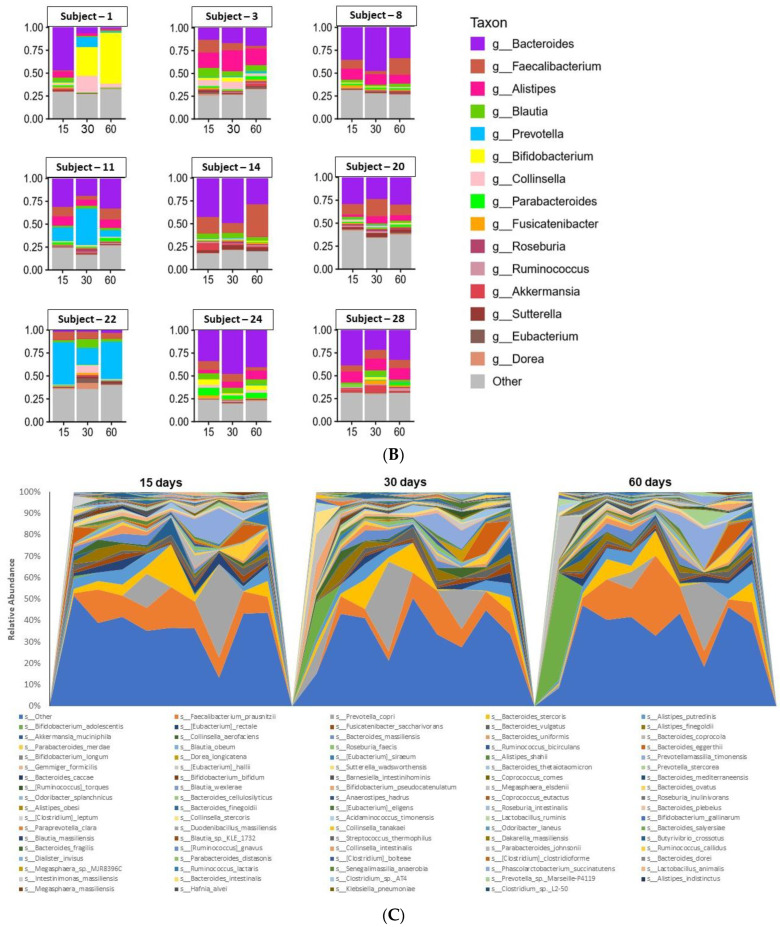
Stack plots illustrating the quantitative distribution of microbial species (**A**) and genus (**B**) of the subjects demonstrating greater resistance to UV irradiation (*n* = 9) on Days 15, 30 and 60. Area charts illustrating the same data are also presented (**C**). Subjects are listed by number in Table 1.

**Figure 3:**
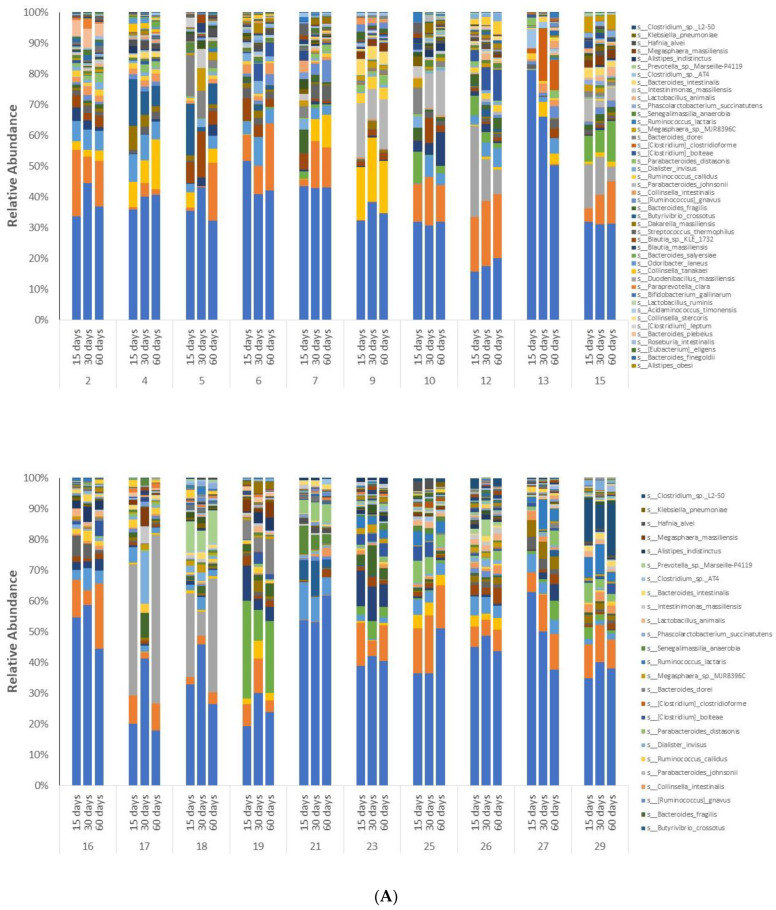

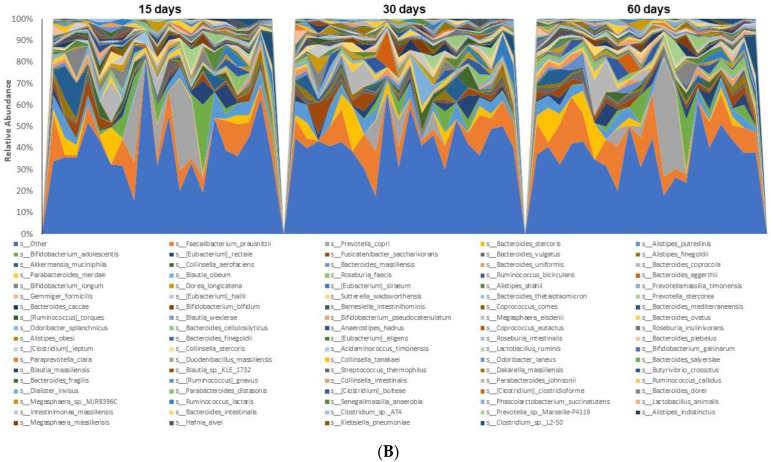
Stacked plots (**A**) and area charts (**B**) presenting diversity of the species for the subjects not resistant to UV irradiation following grape consumption (*n* = 20) on Days 15, 30 and 60. Subjects are listed by number in Table 1.

However, variation in taxonomic abundance was observed with the nine individual subjects on Days 15, 30 and 60. Alterations in species and genera were observed, as illustrated in the stacked plots presented in Figure 2A,B, respectively. Interestingly, with the exception of Subject 1, the extent of variation observed with these nine subjects (Figure 2C) did not appear as profound as with the remaining 20 subjects (Figure 3A,B).

For further characterization, comparative microbiomic taxonomy, enzyme levels, and Kyoto Encyclopedia of Genes and Genomes (KEGG) pathway assessments were performed as follows: Day 15 vs. Day 30; Day 15 vs. Day 60; Day 30 vs. Day 60; with all 29 volunteers; with the nine volunteers responsive on Day 30 (see Table 1); with the three volunteers responsive on Day 30 and Day 60 (see Table 1). In every case, the comparisons demonstrated significant differences (Supplementary File S5). Focusing on the nine subjects, following two weeks of grape consumption, the taxonomic abundance of four genera altered to the greatest extent are listing in Table 2. Although *p*-values adjusted for FDR did not reach the accepted criterion of statistical significance (*q* ≤ 0.05), Cohen’s *d*-values for effect size in the range of 1 suggest an increase in the abundance of *Monoglobus* and *Neglecta*, and decrease in the abundance of *Catonella* and *Holdemania*, in about 85% of the subjects (8 out of 9). Further, with this group of nine responders, the levels of about 15 enzymes were modulated following grape consumption (up to ±13-fold), although KEGG pathway analysis revealed fewer changes, other than oxidoreductases enhanced about 6-fold (Table S1 and Table S2, respectively, Supplementary File S5). Further alterations in each of these three parameters were observed in comparing Day 30 vs. Day 60 (Tables S3–S5, Supplementary File S5), including dramatic reductions in KEGG pathways termed oxidoreductases and extracellular nucleation-precipitation pathway (approximately 4000-fold). Variations were further noted in the Day 15 vs. Day 60 comparisons (Tables S6–S8, Supplementary File S5).

Day 30 comparison of the taxonomic abundance observed with the nine subjects demonstrating UV resistance following grape consumption with the remaining 20 non-responders demonstrated variation in species and genera with *q*-values in the range of 0.1 (Table 3), and many statistically significant variations in comparative enzyme levels (Table 4) and KEGG pathway analysis (Table 5). Interestingly, in essentially every case, abundance and levels were reduced in the nine responders relative to the 20 non-responders. Similarly, comparing the taxa, enzymes and pathways of the nine responders to the 20 non-responders on Day 15 (Tables S9–S11, Supplementary File S5) and Day 60 (Tables S12–S14, Supplementary File S5) revealed significant differences. As was the case on Day 30, essentially all of the parameters assessed were lower in the group of nine relative to the group of 20.

As noted above, of the nine subjects demonstrating enhanced UV resistance on Day 30, three subjects demonstrated enhanced resistance on both Day 30 and Day 60. We were interested in further exploring any unique differences between these two sub-populations, as well as any unique differences between the group of three persistent responders and the entire group of 20 non-responders. First of all, from the outset of the study (Day 15), this group of three demonstrated profound differences relative to the group of 20 non-responders in terms of taxonomy (Table 6). In every case, taxonomic abundance was reduced, which quantitatively varied from about 12-fold to over 140,000-fold lower. This correlated with relatively reduced enzymes levels, many of which were around 50-fold lower (Table S15), and KEGG pathways, in which all of those found to significantly differ were lower, typically around 15-fold (Table S16).

Relative to the group of 20 non-responders, the same patterns persisted on Day 30 and Day 60. With rare exceptions, with the three persistent responders, the abundance of bacterial species that differed in the two groups were in the range of 3- to 700-fold lower (Tables S17 and S20). On Day 30, all enzyme levels that differed in the two groups were reduced, some in the range of 20-fold (Table S18). KEGG pathway analysis correlated with this trend. Every differential pathway identified was reduced, ranging from about 2- to 30-fold (Table S19). At Day 60, the lower differential trend continued, in terms of taxonomic abundance (Table S20), enzyme levels (Table S21), and KEGG pathway analysis (Table S22).

We were also curious as to how the profiles of the three subjects resistant on Day 30 and 60 compare with the remaining six subjects who were resistant only on Day 30. From the outset (Day 15), as was the case when comparing with the group of 20, relative to the group of six, taxonomic abundance of every species showing a difference was reduced, some by as much as about 955-fold (Table S23). Differences in enzyme levels were numerous but, unlike the comparison with the group of 20 non-responders, relative levels varied, although the highest increase was around 2.5-fold, whereas the largest decrease was around 55-fold (Table S24). In terms of KEGG pathway analysis, relative to the comparison with the 20 non-responders, there were fewer differences, several of which were modest increases (e.g., about 1.5-fold) (Table S25). Nonetheless, three pathways were reduced (up to about 7-fold), as compared with 11 major pathway reductions relative to the 20 non-responders (Table S16). With the exception of some KEGG pathways, *q*-values for these latter analyses typically fell in the range of 0.05–0.1, but the trends were apparent.

On Day 30, taxonomic differences remained, but were greatly tempered relative to Day 15 (Table S26). Differences in enzyme levels remained numerous and varied, both elevated and reduced (Table S27). Pathway analysis did not reveal great differences, although those showing significant variation generally trended downward around 2-fold (Table S28).

Comparative taxonomic analysis for Day 60, when the three subjects continued to show UV resistance, whereas the other six did not, is shown in Table 7. Although differences exist, they are mild, still trending downward, but none greater than about 2.5-fold. The same applied for relative enzyme levels, again, with any variations being in the range of about 2-fold (Table 8). Comparative KEGG pathway analysis showed many variations, but again tempered, with greatest differences in the range of about 5-fold lower (Table 9).

Next, to explore some possible ramifications of the dissimilarities described above, metabolomic analyses were undertaken with plasma and urine samples obtained from the volunteers on Day 15, 30 and 60. The following molecules were identified by GC-MS with urine, quantitated relative to an internal standard (4-chlorophenylacetic acid) and analyzed using SIMCA 17 for multivariate data analysis and in GraphPad Prism (v. 9.4.1) for univariate analysis using the Mann–Whitney test (nonparametric unpaired *t*-test): Tartaric acid, erythritol, carbamic acid, 5-hydroxy3-indoleacetic acid, 2-aminomalonic acid, gluconic acid, malonic acid, 3-indoleacetic acid, glycolic acid, glucose, galactose, (2*R*,3*S*)-dihydroxybutanoic acid, 5-oxoproline, lactic acid, threonic acid, *myo*-inositol, fumaric acid, creatinine, palmitic acid, glutaconic acid, lactose, glycine, citric acid, erythronic acid, valine, 2′-deoxyribonic acid, ribitol, 3-hydroxyisovaleric acid, threonine, alanine and ribonic acid. The following molecules were identified by GC-MS with plasma: 3-Hydroxybutanoic acid, urea, 1,5-anhydroglucitol, glucose, galactose, glycolic acid, glyceric acid, pentadecanoic acid, palmitoleic acid, *N*-acetylglycine, methyl-β-D-galactoside, erythritol, levoglucosan, proline, palmitic acid, oleic acid, fructose, nonanoic acid and 2-*O*-glycerol-α-D-galactoside.

**Figure 4:**
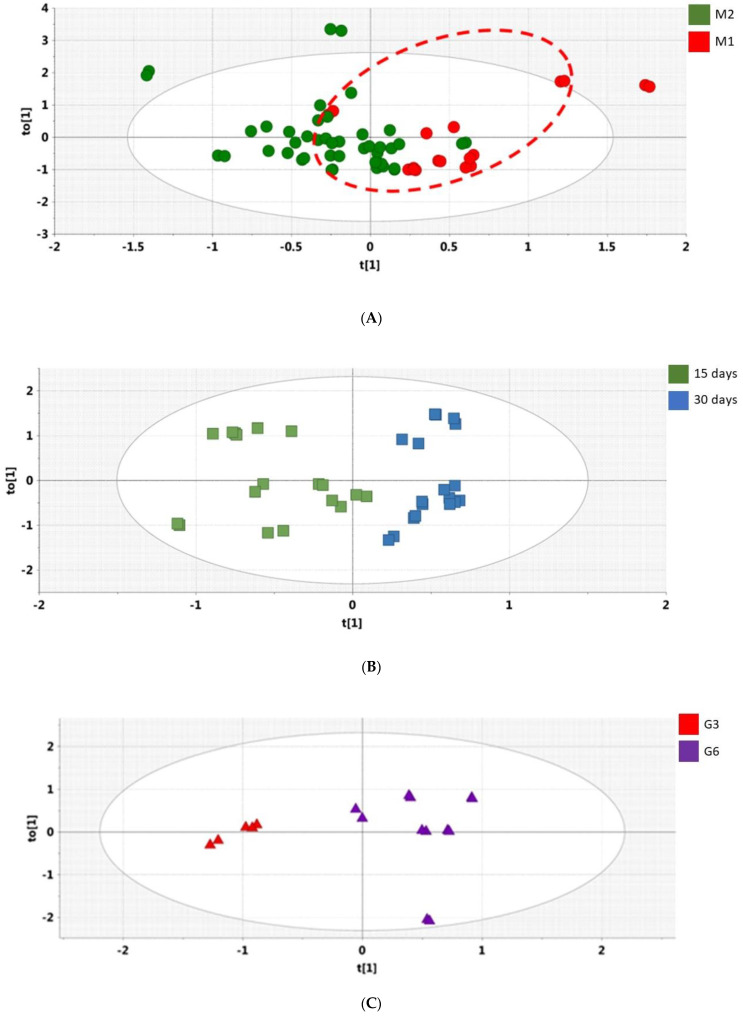
(**A**), OPLS-DA Scores plot for Day 15 urines vs. Day 30 urines showing UV-resistant subjects (M1; *n* = 9; red) and non-resistant subjects (M2; *n* = 20; green). Data presented as duplicate analyses; (**B**), OPLS-DA Scores plot for Day 15 urines (green) vs. Day 30 urines (blue) for UV-resistant subjects (*n* = 9). Data presented as duplicate analyses; (**C**), Day 60 OPLS-DA scores plot for urine showing separation of subgroup G3 (*n* = 3; red) vs. subgroup G6 (*n* = 6; purple). G3 showed UV resistance on Day 30 and Day 60; G6 showed UV resistance on Day 30, but not Day 60. The difference is still apparent after the final washout period (Day 60). Data presented as duplicate analyses.

**Figure 5:**
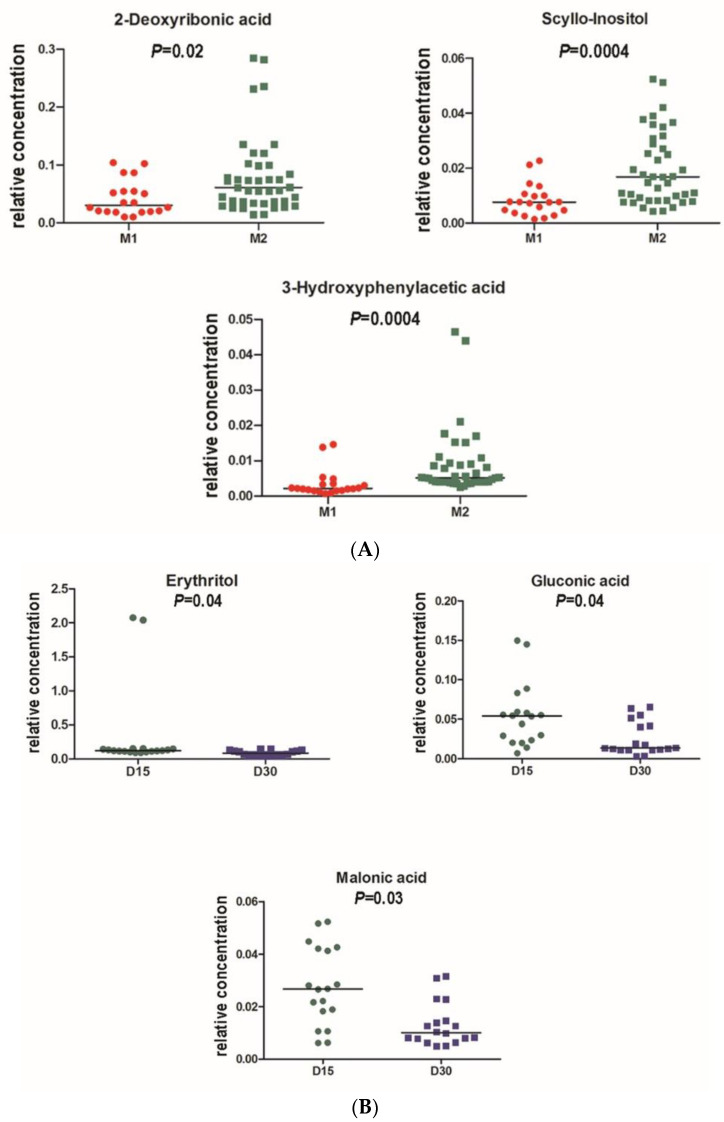

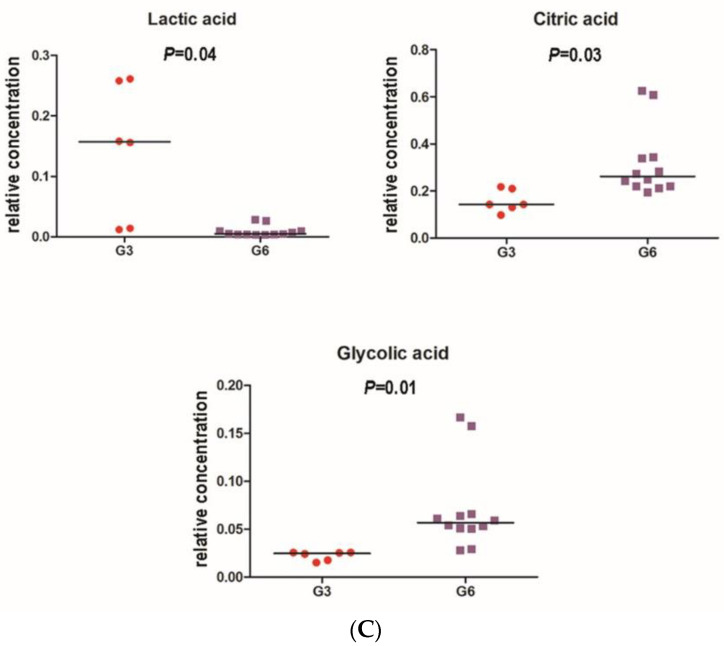
(**A**), Univariate analysis (Mann–Whitney) for a grape diet (Day 30) compared to restricted diet (Day 15) for UV-resistant subjects (M1; *n* = 9; red) and non-resistant (M2; *n* = 20; green) subjects. Data presented as duplicate analyses; *p*-values are Bonferroni corrected for four comparisons. All three urinary metabolites shown were depressed in the UV-resistant group. (**B**), Univariate analysis (Mann–Whitney) for urinary metabolites after grape diet (Day 30; blue) compared to restricted diet (Day 15; green) for UV-resistant subjects (*n* = 9). Data presented as duplicate analyses; *p*-values are Bonferroni corrected for 14 comparisons and remain barely statistically significant. All three urinary metabolites shown were depressed by grape diet. (**C**), Univariate analysis (Mann–Whitney) for urinary metabolites after the final washout period (Day 60) showing separation of subgroup G3 (*n* = 3; red) vs. subgroup G6 (*n* = 6; purple). G3 showed UV resistance on Day 30 and Day 60; G6 showed UV resistance on Day 30 but not Day 60. Data presented as duplicate analyses; *p*-values are Bonferroni corrected for 4 comparisons.

Figure 4A shows the OPLS-DA scores plot for Day 15 vs. Day 30 obtained with the 24 h urine samples obtained from the 20 non-responders (green) and the nine responders (red). The groups are clearly differentiated, and rigorous statistical analysis revealed three urinary metabolites, 2′-deoxyribonic acid, 3-hydroxyphenyl acetic and *scyllo*-inositol, were depressed in the UV-resistant group relative to the remaining 20 subjects (Figure 5A). OPLS-DA scores plot obtained with 24 h urine specimens of the nine responders on Day 15 vs. Day 30 (Figure 4B) revealed depression of erythritol, gluconic acid, and malonic acid (Figure 5B) induced by the grape diet. OPLS-DA scores plot of the three responders on Day 60, relative to the six responders on Day 30, but not Day 60 (Figure 4C), again showed separation, in which the group of three showed enhanced lactic acid and depressed citric and glycolic acids (Figure 5C).

Similar to the above analyses conducted with urine samples derived from the subject population, additional analyses were performed with plasma samples collected during the same time periods. In the Day 15 vs. 30 comparison of the nine responders with the remaining 20 non-responding subjects, separation could be discerned, but statistical significance for metabolites was not obtained (Figure S3, Supplementary File S6). Day 15 vs. Day 30 comparison performed with the nine subjects resistant to UV irradiation on Day 30 showed significant separation, which indicating a change induced by grape consumption (Figure S4, Supplementary File S6). In this case, the level of plasma urea was found to be reduced (Figure S5, Supplementary File S6).

**Figure 6:**
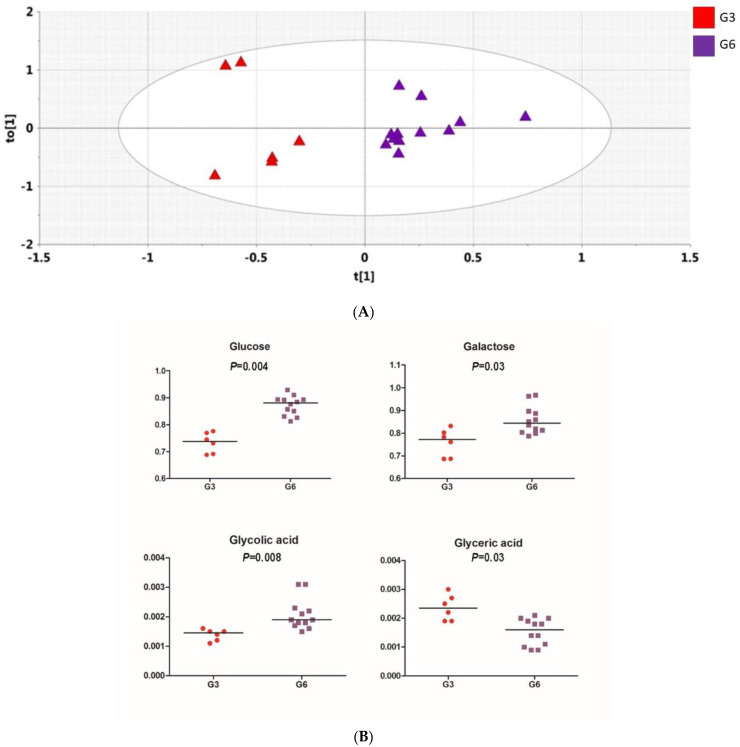
(**A**) OPLS-DA scores plot for plasma showing separation of subgroup G3 (*n* = 3; red) vs. subgroup G6 (*n* = 6; purple). G3 showed UV resistance on Day 30 and Day 60; G6 showed UV resistance on Day 30, but not Day 60. The difference is still apparent after the final washout period (Day 60). Data presented as duplicate analyses. (**B**) Univariate analysis (Mann–Whitney) for plasma metabolites after the final washout period (Day 60) showing separation of subgroup G3 (*n* = 3; red) vs. subgroup G6 (*n* = 6; purple). Data presented as duplicate analyses; *p*-values are Bonferroni corrected.

Of particular interest was a comparison of the three individuals who were resistant to UV irradiation on Day 60 as well as Day 30, with the remaining six individuals who demonstrated resistance only on day 30. As shown in the OPLS-DA scores plot illustrated in Figure 6A, on Day 60, these three individuals could be clearly differentiated from the remaining six who were responsive on Day 30, but not on Day 60. Further, as shown in Figure 6B, relative to the six individuals no longer showing resistance on Day 60, the three responders on Day 60 demonstrated reduced plasma levels of glucose, galactose, and glycolic acid, and an elevated level of glyceric acid.

**Figure 7:**
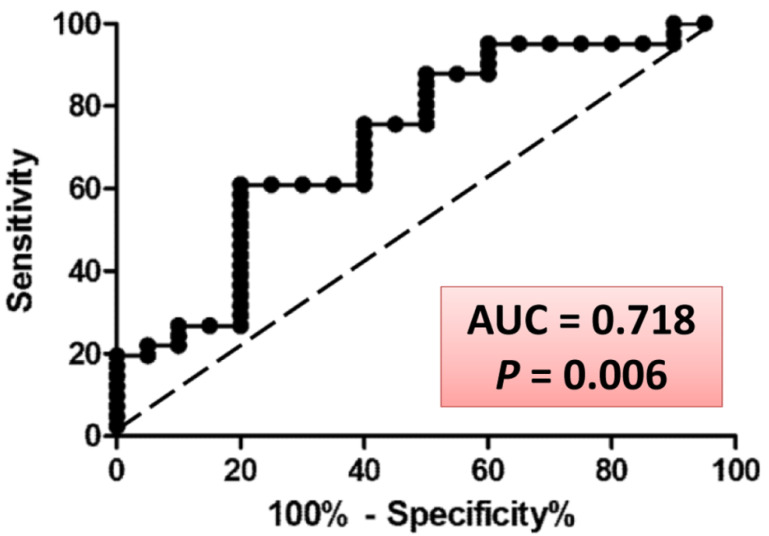
ROC curve for the effect of grape consumption on urinary 2′-deoxyribonic acid (responders vs. non-responders for UV skin MED). There is a 71.8% probability (*p* = 0.006) that measurement of urinary 2′-deoxyribonic acid identifies an individual who will not exemplify greater resistance to UV irradiation following grape consumption.

Finally, of special interest was 2′-deoxyribonic acid, a known by-product of UV-induced damage of DNA [43]. As noted above, the quantity of this substance was reduced in urine specimens collected from volunteers who demonstrated enhanced resistance to UV irradiation. The mass spectrum of a peak eluting at 21.51 min from pooled urine of subjects in this study was compared to the partial published spectra deriving from both the DNA sugar damage product and authentic 2′-deoxyribonic acid as their tetrasilyl derivatives [44]. All three mass spectra were virtually identical, with major ions at *m*/*z* 189, 201, 217, 233, 245, 259, 306, 321 and 335, confirming the structure of our urinary metabolite as 2′-deoxyribonic acid. To investigate this further, a ROC curve for the effect of grape diet on urinary 2′-deoxyribonic acid (responders vs. non-responders for UV skin MED) was constructed (Figure 7). Based on this analysis, it can be deduced that measurement of urinary 2′-deoxyribonic acid identifies a UV skin non-responder with a 71.8% probability (*p* = 0.006).

## 4. Discussion

A key object of this study was to assess the potential of dietary grapes to enhance resistance to UV irradiation of the skin as judged by MED determinations. Conforming to best practice standards [45], a standardized product equivalent to three daily servings of fresh grapes was consumed by human volunteers for a period of two weeks. In addition, dietary, medication, and lifestyle restricts were imposed and found acceptable by all volunteers during the study period. As a result, with a group of 29 subjects who completed the study protocol, nine (31%) were found to demonstrate significantly greater resistance to UV irradiation following grape consumption. This confirms and expands the investigations of Elmets et al., in which enhanced resistance was reported in 11 of 19 subjects (57%). We consider our results to be completely consistent with those reported previously [13,14]. The same protocol was utilized for the consumption of grapes, although there was some variation in UV treatment procedures. Perhaps of greatest importance was the variation of the Fitzpatrick skin types of the subjects participating in the respective trials. Our study included five subjects with Type II (17%) and the remaining 24 subjects with Type III (83%), whereas the Elmets study included two subjects with Type I (10%), 12 subjects with type II (63%), and five subjects with Type III (26%). As such, the results of the two trials are extremely complementary. Therefore, we conclude that dietary consumption of grapes does enhance resistance to UV irradiation of the skin in a significant portion of the population, and the extent of responsiveness may be greater with individuals with lower Fitzpatrick skin types. Based on our results in conjunction with the results of Elmets et al. [13,14], we have no valid reason for concluding that a positive response varies with age or gender. Both males and females are responsive, over an age range spanning > 30 years.

Considering these results, we and others are curious about what attributes may be unique in those subjects who are responsive as a result of grape ingestion, and those who are not responsive. First, each volunteer was subjected to a thorough analysis at the UV irradiation site based on the Commission Internationale de l’Eclairage (CIE) L*a*b* color scale. The continuity of these results strongly suggests there was no bias in outcome due to skin attributes. Particularly noteworthy, the group of nine subjects who demonstrated enhanced resistant to UV irradiation showed no differences relative to the remaining 20 subjects participating in the study. The only change that was observed in the L*a*b* color scale during the entire course of the study was an increase in L* that reached statistical significance at Day 60. However, given the low magnitude of this change, visualization of the shades of tan on Day 60 versus earlier time points using a color converter makes it clear there is no meaningful difference. Moreover, since the group of nine subjects who demonstrated enhanced resistance to UV irradiation showed no differentiation in L* relative to the remaining 20 subjects participating in the study, it is seems clear this minor change was not related to the study protocol or the consumption of grapes.

Obviously, however, there is something unique about the nine subjects who demonstrated greater resistance. Elmets and coworkers demonstrated significantly lower levels of cyclobutane pyrimidine dimers and double-strand breaks in DNA volunteers consuming grapes, as well as down-regulation of proinflammatory pathways [14]. In studies conducted with mice, where inter-individual variation is limited, Ahmad and coworkers demonstrated dietary grapes enhanced DNA damage repair, reduced proliferation, increased apoptosis, modulated oxidative stress markers, and decreased mast cell infiltration, serum IgE and Eotaxin [10,11,12]. These data deal directly with the erythema response and are of great interest. In the current study, given that, in our human subject population, nine individuals responded strongly and the remainder showed no appreciable response, we elected to investigate how systemic responses to grape consumption may vary in the two populations.

Considering the wide-ranging appreciation for the potential of diet to influence the microbiome [46,47], the broad and profound impact of the microbiome on human health and well-being [48,49], and, in particular, an increasing appreciation of the gut–skin axis [50], we compared the relative composition and abundance of the microbiota in these two populations. From the outset of the study (Day 15), differences in taxonomic, enzymic and pathway analyses were striking. Many significant differences persisted throughout the duration of the study, primarily reflected in reduced microbial abundance and the ramifications thereof in the UV resistant populations. While it is possible to speculate on the biological and physiological impact of these differences and modulations, a direct glimmer of outcome can be gained through metabolomics.

In previous studies, we have investigated the composition of the microbiome with this entire subject population of 29, as well as metabolomics analyses [16]. In brief, taking the group as a whole, partial least squares-discriminant analysis (PLS-DA) scores plot for Day 15 vs. Day 30 showed little difference with plasma. On Day 60, following termination of grape consumption, some serum sugars were elevated. In urine, with the group as a whole, on Day 30, 2′-deoxyribonic acid, glutaconic acid, and 3-hydroxyphenyl acetic acid were elevated, whereas 3-indoleacetic acid, hippuric acid, valine, ribose, 2,3-dihydroxybutanoic acid, galactose, glucose, carbamic acid, malonic acid, and levoglucosan were reduced.

In the current work, the nine subjects responsive to grape consumption and resistant to UV irradiation were clearly distinguished from the remaining 20 volunteers through metabolomic analyses as well as microbiomic analyses. Notably, as shown in Figure 5A, at Day 30, significant reductions in 2′-deoxyribonic acid, 3-hydroxyphenyl acetic and *scyllo*-inositol were observed with this UV resistant group. We are particularly intrigued by the levels of 2′-deoxyribonic acid being elevated in the group as a whole, but reduced in this select group of nine responders. Although the origin of 2′-deoxyribonic acid may be considered moot [51], and the substance is found in the urine of healthy human beings [51,52], elevated levels are clearly generated by γ-irradiation or cancer chemotherapy [18] and, as demonstrated by Hasiba et al. [43], by UV irradiation of DNA. The resulting DNA lesion is viewed as lethal or mutagenic [53]. Thus, these results suggest less DNA damage, of the skin or otherwise, for this select group with the grape diet. Even moreover, based on a ROC (receiver operating characteristic) curve derived from responders vs. non-responders for UV skin MED, it can be predicted with 71.8% probability (*p *= 0.006) that measurement of urinary 2′-deoxyribonic acid will identify a UV skin non-responder.

It is pertinent to consider the known mechanism by which 2′-deoxyribonic acid is released after DNA radiation damage. It was reported over four decades ago that γ-irradiated DNA in aqueous solution released “2-deoxy-D-erythro-pentonic acid” [(3*S*,4*R*)-3,4,5-trihydroxypentanoic acid; 2′-hydroxyribonic acid] [54]. Free radical attack on deoxycytidylate residues in double-stranded DNA was later found to release two products, demonstrated by HPLC and GC-MS, to be 2′-deoxyribonic acid and its lactone, 2′-deoxyribonolactone. The mechanism proposed was the abstraction of a hydrogen to form a carbon-centered radical at the C-1′ of deoxyribose, and subsequent oxidation at this position yielding the observed products [44], which we detect as tetrasilylated 2′-deoxyribonic acid by GC-MS. It was also demonstrated that UV irradiation generated this same product at ACA mutagenic hotspots using the synthetic trinucleotide d(ApCpA). The deoxyribose that had been attached to the eliminated cytosine was ring-opened to a 2′-deoxyribonic acid moiety, which was proposed as responsible for the mutagenic potential of the ACA hotspot due to the resistance of cleavage by certain endonucleases [53]. The mechanism of oxidative damage to the DNA backbone by abstraction of hydrogen from the C-1′ position of deoxyribose, leading to production of 2′deoxyribonolactone in situ, was subsequently confirmed using various DNA structures [43].

In sum, UV irradiation is able to form and release 2′deoxyribonic acid and its lactone from DNA. It is well established that the response of the skin to UV irradiation involves production of reactive oxygen species [55], which is undoubtedly responsible for the oxidation of the DNA backbone to release 2′deoxyribonolactone. The question remains, why does the erythema response, which is associated with greater urinary excretion of 2′-deoxyribonic acid, occur in some individuals and not in others? Such variation in skin response to PUVA therapy (UV A irradiation combined with oral 8-methoxypsoralen administration) is well known to dermatologists [56]. The glutathione *S*-transferase genes GSTM1, GSTT1 and GSTP1 are polymorphic with null alleles for GSTM1 and GSTT1 arising from complete gene deletions associated with an absence of enzyme function that is inherited by 50% and 20% of Caucasians, respectively [57,58]. The GSTM1 and GSTT1 polymorphisms influence the cutaneous sensitivity to UV radiation [59]. It is therefore likely that the observed differences in skin reaction to UV light reported here are due to these common polymorphisms in glutathione *S*-transferase activity and mirrored in variable urinary excretion of the DNA damage product 2′-deoxyribonic acid. Future investigations in this field will require determination of GSTM1 and GSTT1 genotypes to clarify this point.

Yet, another interesting aspect of this study was that three of the nine subjects who acquired UV resistance after two weeks of grape consumption retained this characteristic of resistance 30 days after ceasing grape consumption. The remaining six subjects did not show resistance on Day 60, which afforded us the opportunity to compare these two groups. A variety of significant differences were discerned in terms of microbiological taxonomy, enzymes, pathways and metabolomics, relative to the group as a whole, as well as relative to the six individuals who were responsive to grape consumption on Day 30 but not on Day 60. Compared with the latter group of six, on Day 60, glucose, galactose and glycolic acid were reduced in plasma, and glyceric acid was elevated in plasma. In urine, lactic acid was elevated, and citric acid and glycolic acid were reduced.

Finally, in the context of grape consumption, it seems rational to reflect on what can be viewed as cause-and-effect with any reasonable degree of credibility. On one hand, it is necessary to employ an “omics” approach to gain a more realistic understanding of dietary response on a holistic level. On the other hand, interpretation of the biological and physiological ramifications of a tsunami of data becomes challenging. Nonetheless, it seems fair to conclude, in conjunction with the results reported by Elmets [13,14], that a certain segment of the population will demonstrate greater resistance to dermal response elicited by UV irradiation as a result of grape consumption. Further, our results suggest that individuals who are capable of mounting this response have unique microbiomic and metabolomic characteristics that correlate with the ability to acquire UV resistance. It is likely that many of the numerous differences we report comparing those who show enhanced resistance to UV irradiation as a result of grape consumption, and those who do not respond, either before, during or after grape consumption, are not essential for this trait of UV resistance. However, it is likely that some grape-induced alterations are required, given that grape consumption leads to the response. It is rational to suggest the microbiome is involved in some way, given our increasing appreciation for the gut-skin axis. More broadly, it is reasonable to expect other ramifications in regard to health, not only with the nine volunteers who showed increased UV resistance, but with others as well, taking into account the gut-brain, gut-liver, gut-lung, gut-immune, gut-etc. axes. A great deal of additional research will be necessary to test this supposition.

## Figures and Tables

**Table 1:** Subject demographics, skin type, and change in minimal erythema dose (MED) response.

Subject	Age	Sex	Race	Ethnicity	Fitzpatrick	ΔMED ^3^	
					Skin-Type	Day 30	Day 60
1	24.0	Male	White/Caucasian	Non-Hispanic/Latino	III ^1^	+9.90	0
2	30.0	Male	White/Caucasian	Non-Hispanic/Latino	III	0	0
3	37.0	Female	White/Caucasian	Hispanic/Latino	III	+12.40	+12.40
4	43.3	Female	White/Caucasian	Non-Hispanic/Latino	III	0	0
5	45.3	Female	White/Caucasian	Non-Hispanic/Latino	III	0	−6.30
6	45.3	Female	White/Caucasian	Non-Hispanic/Latino	III	0	0
7	32.6	Female	White/Caucasian	Hispanic/Latino	III	0	0
8	33.9	Male	White/Caucasian	Non-Hispanic/Latino	III	+8.00	+8.00
9	29.4	Female	White/Caucasian	Non-Hispanic/Latino	III	0	0
10	40	Male	White/Caucasian	Hispanic/Latino	III	0	0
11	33.6	Male	White/Caucasian	Non-Hispanic/Latino	III	+9.90	+9.90
12	44.4	Male	White/Caucasian	Non-Hispanic/Latino	II ^2^	0	0
13	34.9	Female	White/Caucasian	Hispanic/Latino	III	0	0
14	34.8	Female	White/Caucasian	Non-Hispanic/Latino	III	+9.90	0
15	39.0	Female	White/Caucasian	Hispanic/Latino	III	0	−9.90
16	44.3	Female	White/Caucasian	Non-Hispanic/Latino	III	0	0
17	36.2	Female	White/Caucasian	Non-Hispanic/Latino	III	0	0
18	42.9	Female	White/Caucasian	Hispanic/Latino	III	0	0
19	43.2	Male	White/Caucasian	Non-Hispanic/Latino	III	0	0
20	43.7	Male	White/Caucasian	Hispanic/Latino	III	+8.00	0
21	52.9	Female	White/Caucasian	Hispanic/Latino	III	0	0
22	46.8	Male	White/Caucasian	Non-Hispanic/Latino	III	+6.30	0
23	51.6	Male	White/Caucasian	Non-Hispanic/Latino	II	0	−6.30
24	54.4	Male	White/Caucasian	Non-Hispanic/Latino	II	+6.30	0
25	48.2	Male	White/Caucasian	Non-Hispanic/Latino	III	0	0
26	37.7	Male	White/Caucasian	Non-Hispanic/Latino	III	0	0
27	55.7	Male	White/Caucasian	Non-Hispanic/Latino	II	0	0
28	46.1	Male	White/Caucasian	Non-Hispanic/Latino	III	+9.90	0
29	55.1	Male	White/Caucasian	Non-Hispanic/Latino	III	0	0

^1^ Type III—Burns moderately, tans gradually; ^2^ Type II—Always burns easily, tans gradually; ^3^ ΔMED, mJ.

**Table 2:** Comparison of the taxonomy of Day 15 vs. Day 30 for subjects resistant to UV irradiation following grape consumption (*n* = 9).

Taxonomy ^1^	Log2 (Fold-Change)	*Q* Value	*D* Value	Functional Connotations
g__*Catonella*	−2.994	0.083	1.013	Associated with *Lachnospiraceae* family. Increases in *Lachnospiraceae* abundances are associated with aging [22].
g__*Holdemania*	−2.168	0.083	0.929	Leads to reduction in the vegetarian diet [23].
g__*Neglecta*	2.082	0.112	1.027	Lead to increase in the abundance with the consumption of glycans [24].
g__*Monoglobus*	2.982	0.224	1.040	Pectin is abundant in modern day diets, as it comprises the middle lamellae and one-third of the dry carbohydrate weight of fruit and vegetable cell walls [25].

^1^ Taxonomic hierarchies are designated as c (class), o (order), f (family), g (genus) or s (species).

**Table 3:** Day 30 comparison of the taxonomic abundance of subjects demonstrating UV resistance following grape consumption (*n *= 9) vs. subjects not resistant to UV irradiation following grape consumption (*n *= 20).

Taxonomy ^1^	Log2 (Fold-Change)	*Q* Value	Functional Connotations
g__*Tannerella*	−1.281	0.074	Associated with periodontal inflammation [26].
g__*Blautia*	−0.916	0.077	Alleviates inflammatory diseases and metabolic diseases [27].
s__*Blautia_massiliensis*	−1.706	0.085	Alleviates inflammatory diseases and metabolic diseases [27].
s__*Ruminococcus_bicirculans*	−2.264	0.087	Degrade dietary cellulosic biomass into nutritive short-chain fatty acids [28].
s__*Blautia_*sp.*_KLE_1732*	−1.841	0.094	Alleviates inflammatory diseases and metabolic diseases [27].
s__*Parabacteroides_distasonis*	−0.765	0.094	Alleviates obesity and metabolic dysfunctions [29].
g__*Intestinibacillus*	−3.203	0.10	Role in counteraction to infectious diseases [30].
s__*Anaerostipes_hadrus*	−1.281	0.11	Increased butyrate content in the gut [31].
g__*Eisenbergiella*	−0.981	0.11	Ketone diet could enrich *Eisenbergiella massiliensis* in human gut [32].
s__*[Clostridium]_leptum*	−2.366	0.13	Maintains the intestinal microecological balance, promotes immune maturation, and increases Treg numbers to alleviate airway inflammation [33].
s__*Bacteroides_dorei*	−1.525	0.14	Higher abundance is linked with Type I diabetes [34].

^1^ Taxonomic hierarchies are designated as c (class), o (order), f (family), g (genus) or s (species).

**Table 4:** Day 30 comparison of the enzyme levels of subjects demonstrating UV resistance following grape consumption (*n *= 9) vs. subjects not resistant to UV irradiation following grape consumption (*n *= 20).

Enzymes	Log2 (Fold-Change)	*Q* Value
1.17.5.3 fdnG; formate dehydrogenase-N, alpha subunit	−2.339	0.027
5.5.1.2 pcaB; 3-carboxy-*cis*,*cis*-muconate cycloisomerase	−1.513	0.027
5.3.3.14 fabM; *trans*-2-decenoyl-[acyl-carrier protein] isomerase	−1.267	0.027
2.1.1.315 rif14; 27-*O*-demethylrifamycin SV methyltransferase	−1.334	0.027
6.5.1.1 ligD; bifunctional non-homologous end joining protein LigD	−1.527	0.028
3.4.21.62 aprE; subtilisin	−3.564	0.028
3.2.1.22 melA; alpha-galactosidase	−1.378	0.029
1.8.5.4 sqr; sulfide:quinone oxidoreductase	−1.297	0.029
2.1.1.265 tehB; tellurite methyltransferase	−1.874	0.029
1.5.1.24 ceo; *N*5-(carboxyethyl)ornithine synthase	−4.532	0.029
2.7.13.3 cpxA; two-component system, OmpR family, sensor histidine kinase CpxA	−1.211	0.030
1.8.4.10 cysH; phosphoadenosine phosphosulfate reductase	−1.865	0.030
1.8.4.8 cysH; phosphoadenosine phosphosulfate reductase	−1.865	0.030
6.3.2.14 entE, dhbE, vibE, mxcE; 2,3-dihydroxybenzoate-AMP ligase	−3.328	0.031
4.2.1.167 hgdA; (*R*)-2-hydroxyglutaryl-CoA dehydratase subunit alpha	1.212	0.031
2.3.1.197 ftdC; dTDP-3-amino-3,6-dideoxy-alpha-D-galactopyranose 3-*N*-acetyltransferase	−1.387	0.038
2.7.7.58 entE, dhbE, vibE, mxcE; 2,3-dihydroxybenzoate-AMP ligase	−3.328	0.038
2.4.1.60 rfbV; abequosyltransferase	−3.127	0.042
5.3.1.22 hyi, gip; hydroxypyruvate isomerase	−1.407	0.049

**Table 5:** Day 30 comparison of the Pathways (KO level 3) of subjects demonstrating UV resistance following grape consumption (*n *= 9) vs. subjects not resistant to UV irradiation following grape consumption (*n *= 20).

Pathways	Log2 (Fold-Change)	*Q* Value
1. Oxidoreductases; 1.7 Acting on other nitrogenous compounds as donors; 1.7.2 With a cytochrome as acceptor	−1.119	0.038
1. Oxidoreductases; 1.3 Acting on the CH-CH group of donors; 1.3.99 With other acceptors	−0.259	0.039
3. Hydrolases; 3.4 Acting on peptide bonds (peptidases); 3.4.17 Metallocarboxypeptidases	0.456	0.039
1. Oxidoreductases; 1.7 Acting on other nitrogenous compounds as donors; 1.7.99 With other acceptors	−0.437	0.040
Nonribosomal peptide synthetase (NRPS); Iterative NRPS; Bacillibactin synthetase	−3.191	0.042
OmpR family; BasS-BasR	−1.257	0.043
OmpR family; PhoR-PhoB (phosphate)	−0.290	0.045
OmpR family; CpxA-CpxR	−0.998	0.046
ABC Transporters, Prokaryotic Type; Monosaccharide transporters; Ribose transporter [MD:M00212]	−0.703	0.048
ABC Transporters, Prokaryotic Type; Metallic cation, iron-siderophore and vitamin B12 transporters; Manganese transporter [MD:M00316]	−0.793	0.050
Nonribosomal peptide synthetase (NRPS); Nonlinear NRPS; Vibriobactin synthetase	−3.388	0.051

**Table 6:** Day 15 comparison of the taxonomic abundance of subjects demonstrating UV resistance following grape consumption on Days 30 and 60 (*n *= 3) vs. subjects not resistant to UV irradiation following grape consumption (*n *= 20).

Taxonomy ^1^	Log2 (Fold-Change)	*Q* Value	Functional Connotations
s__*Bacteroides_dorei*	−3.881	0.030	Higher abundance is linked with Type I diabetes [34].
g__*Barnesiella*	−3.315	0.030	Commensals reduced Treg cells in the tumor microenvironment (Foxp3 and/or γδT17 cells) [35].
s__*Prevotella_copri*	−17.131	0.030	Associated to colitis in mice, exacerbates intestinal inflammation [36].
f__*Prevotellaceae*	−5.163	0.034	Associated to colitis in mice, exacerbates intestinal inflammation [36].
g__*Prevotella*	−7.316	0.036	Associated to colitis in mice, exacerbates intestinal inflammation [36].
f__*Barnesiellaceae*	−3.292	0.037	Commensals reduced Treg cells in the tumor microenvironment (Foxp3 and/or γδT17 cells) [35].
g__*Catonella*	−4.687	0.047	Reside in the oral mucosa as commensals but may be opportunistic pathogens with potential correlations with oral squamous cell carcinoma (OSCC) [37].
s__*Clostridium_*sp.*_AT4*	−4.576	0.047	An increased abundance of *Clostridiaceae* was shared by both inflammatory bowel disease (IBD)-A and rheumatoid arthritis (RA) patients [38,39].
g__*Ruminiclostridium*	−3.925	0.048	Consistently present in the healthy human gut [28].
s__*Barnesiella_intestinihominis*	−3.582	0.050	Commensals reduced Treg cells in the tumor microenvironment (Foxp3 and/or γδT17 cells) [35].

^1^ Taxonomic hierarchies are designated as c (class), o (order), f (family), g (genus) or s (species).

**Table 7:** Day 60 comparison of the taxonomic abundance of subjects demonstrating UV resistance following grape consumption on Days 30 and 60 (*n *= 3) vs. subjects demonstrating resistance to UV irradiation following grape consumption only on Day 30 (*n *= 6).

Taxonomy ^1^	Log2 (Fold-Change)	*Q* Value	Functional Connotations
f_*Acidaminococcaceae*	−1.267	0.018	Found to be higher in disease-related groups [40].
s_*Streptococcus_thermophilus*	0.643	0.020	Anti-inflammatory potential in colitis [41].
o_Acidaminococcales	−1.267	0.027	Found to be higher in disease-related groups [40].
s_*Clostridium_*sp._AT4	−1.188	0.039	Found to be higher in IBD [42].
g__*Acidaminococcus*	−1.139	0.048	Found to be higher in disease-related groups [40].

^1^ Taxonomic hierarchies are designated as c (class), o (order), f (family), g (genus) or s (species).

**Table 8:** Day 60 comparison of the enzyme levels of subjects demonstrating UV resistance following grape consumption on Days 30 and 60 (*n *= 3) vs. subjects demonstrating resistance to UV irradiation following grape consumption only on Day 30 (*n *= 6).

Enzymes	Log2 (Fold-Change)	*Q* Value
3.5.3.1 E3.5.3.1, rocF, arg; arginase	1.031	0.011
3.1.1.17 E3.1.1.17, gnl, RGN; gluconolactonase	0.845	0.011
2.7.13.3 cqsS; two-component system, CAI-1 autoinducer sensor kinase/phosphatase CqsS	0.683	0.011
5.1.3.20 gmhD, rfaD; ADP-L-glycero-D-manno-heptose 6-epimerase	−0.867	0.022
3.4.21.50 E3.4.21.50; lysyl endopeptidase	0.723	0.022
3.5.1.87 pydC; beta-ureidopropionase/*N*-carbamoyl-L-amino-acid hydrolase	−1.164	0.037
2.4.1.317 tylN; *O*-mycaminosyltylonolide 6-deoxyallosyltransferase	0.950	0.038
2.4.2.44 mtiP; 5′-methylthioinosine phosphorylase	−1.080	0.039
3.5.1.6 pydC; beta-ureidopropionase/*N*-carbamoyl-L-amino-acid hydrolase	−1.164	0.041
1.7.1.7 E1.7.1.7, guaC; GMP reductase	−0.715	0.041
3.5.4.16 folE2; GTP cyclohydrolase IB	−0.735	0.048

**Table 9:** Day 60 comparison of pathways (KO level 3) of subjects demonstrating UV resistance following grape consumption on Days 30 and 60 (*n *= 3) vs. subjects demonstrating resistance to UV irradiation following grape consumption only on Day 30 (*n *= 6).

Pathways	Log2 (Fold-Change)	*Q* Value
1. Oxidoreductases; 1.14 Acting on paired donors, with O_2_ as oxidant and incorporation or reduction of oxygen. The oxygen incorporated need not be derived from O_2_; 1.14.19 With oxidation of a pair of donors resulting in the reduction of O_2_ to two molecules of water	0.897	0.044
NarL family; RcsC-RcsD-RcsB	1.165	0.048
2. Transferases; 2.7 Transferring phosphorus-containing groups; 2.7.9 Phosphotransferases with paired acceptors	−1.215	0.053
5. Isomerases; 5.3 Intramolecular oxidoreductases; 5.3.4 Transposing S-S bonds	−2.455	0.055
Non-ion channels; Aquaglyceroporins or glycerol-uptake facilitators	1.003	0.055
ABC Transporters, Prokaryotic Type; Mineral and organic ion transporters; Putrescine transporter [MD:M00300]	0.958	0.056
5. Isomerases; 5.3 Intramolecular oxidoreductases; 5.3.99 Other intramolecular oxidoreductases	1.199	0.057

## Data Availability

The datasets generated and analyzed for the current study are available in the National Center for Biotechnology Information (NCBI) repository, Accession Number PRJNA882649.

## Supplementary Materials

The following supporting information can be downloaded at: https://www.mdpi.com/article/10.3390/antiox11122372/s1, Supplementary File S1, Inclusion/Exclusion Criteria; Supplementary File S2, Subject Responsibilities; Supplementary File S3, Dosing Protocol for Grape Powder; Supplementary File S4, Figure S1: Alpha-diversity; Supplementary File S4, Figure S2. Analysis of beta-diversity was conducted with the 9 subjects found to demonstrate UV resistance following consumption of grapes for two weeks. Cluster analysis (PCA and PCoA plots) revealed no significant differences on Day 15, 30 or 60; Supplementary File S5, Comparative Taxonomy, Enzyme Levels, and KEGG Orthology. Table S1. Comparative enzyme levels determined by comparison of Day 15 vs. Day 30 for the subjects resistant to UV irradiation on Day 30 (*n *= 9); Table S2. KEGG pathways altered when comparing Day 15 vs. 30 with the group resistant to UV irradiation on Day 60 (*n *= 9); Table S3. Taxonomic comparison on Day 30 vs. 60 days for the group resistant to UV irradiation on Day 30 (*n *= 9); Table S4. Comparative enzyme levels determined by comparison of Day 30 vs. Day 60 for the subjects resistant to UV irradiation on Day 30 (*n *= 9); Table S5. KEGG pathways altered when comparing Day 30 vs. 60 with the group resistant to UV irradiation on Day 60 (*n *= 9); Table S6. Taxonomic comparison on Day 15 vs. Day 60 for the group resistant to UV irradiation on Day 30 (*n *= 9); Table S7. Comparative enzyme levels determined by comparison of Day 15 vs. Day 60 for the subjects resistant to UV irradiation on Day 30 (*n *= 9); Table S8. KEGG pathways altered when comparing Day 15 vs. 60 with the group resistant to UV irradiation on Day 60 (*n *= 9); Table S9. Taxonomic comparison of the group resistant to UV irradiation on Day 60 (*n *= 9) with the group of non-responsive subjects (*n *= 20) on Day 15; Table S10. Comparative enzyme levels determined by comparing group resistant to UV irradiation on Day 60 (*n *= 9) with the group of non-responsive subjects (*n *= 20) on Day 15; Table S11. KEGG pathways altered (KO level 3) when comparing group resistant to UV irradiation on Day 60 (*n *= 9) with the group of non-responsive subjects (*n *= 20) on Day 15; Table S12. Taxonomic comparison of the group resistant to UV irradiation on Day 60 (*n *= 9) with the group of non-responsive subjects (*n *= 20) on Day 60; Table S13. Comparative enzyme levels determined by comparing group resistant to UV irradiation on Day 60 (*n *= 9) with the group of non-responsive subjects (*n *= 20) on Day 60; Table S14. KEGG pathways altered (KO level 3) when comparing group resistant to UV irradiation on Day 60 (*n *= 9) with the group of non-responsive subjects (*n *= 20) on Day 60; Table S15. Comparative enzyme levels determined when comparing group resistant to UV irradiation on Day 30 and Day 60 (*n *= 3) with the group of non-responsive subjects (*n *= 20) on Day 15; Table S16. KEGG pathways altered (KO level 3) when comparing group resistant to UV irradiation on Day 30 and Day 60 (*n *= 3) with the group of non-responsive subjects (*n *= 20) on Day 15; Table S17. Taxonomic comparison of the group resistant to UV irradiation on Day 30 and Day 60 (*n *= 3) with the group of non-responsive subjects (*n *= 20) on Day 30; Table S18. Comparative enzyme levels determined when comparing group resistant to UV irradiation on Day 30 and Day 60 (*n *= 3) with the group of non-responsive subjects (*n *= 20) on Day 30; Table S19. KEGG pathways altered (KO level 3) when comparing group resistant to UV irradiation on Day 30 and Day 60 (*n *= 3) with the group of non-responsive subjects (*n *= 20) on Day 30; Table S20. Taxonomic comparison of the group resistant to UV irradiation on Day 30 and Day 60 (*n *= 3) with the group of non-responsive subjects (*n *= 20) on Day 60; Table S21. Comparative enzyme levels determined when comparing group resistant to UV irradiation on Day 30 and Day 60 (*n *= 3) with the group of non-responsive subjects (*n *= 20) on Day 60; Table S22. KEGG pathways altered (KO level 3) when comparing group resistant to UV irradiation on Day 30 and Day 60 (*n *= 3) with the group of non-responsive subjects (*n *= 20) on Day 60; Table S23. Day 15 taxonomic analysis comparing the group resistant to UV irradiation on Day 30 and Day 60 (*n *= 3) with the remaining group subjects responsive only on Day 30 (*n *= 6); Table S24. Day 15 comparative enzyme levels when comparing the group resistant to UV irradiation on Day 30 and Day 60 (*n *= 3) with the remaining group of subjects responsive only on Day 30 (*n *= 6); Table S25. Day 15 analysis of KEGG pathways altered (KO level 3) when comparing the group resistant to UV irradiation on Day 30 and Day 60 (*n *= 3) with the remaining group of subjects responsive only on Day 30 (*n *= 6); Table S26. Day 30 taxonomic analysis comparing the group resistant to UV irradiation on Day 30 and Day 60 (*n *= 3) with the remaining group of subjects responsive only on Day 30 (*n *= 6); Table S27. Day 30 comparative enzyme levels when comparing the group resistant to UV irradiation on Day 30 and Day 60 (*n *= 3) with the remaining group of subjects responsive only on Day 30 (*n *= 6); Table S28. Day 30 analysis of KEGG pathways altered (KO level 3) when comparing the group resistant to UV irradiation on Day 30 and Day 60 (*n *= 3) with the remaining group of subjects responsive only on Day 30 (*n *= 6); Supplementary File S6, Figure S3. OPLS-DA Scores plot for Day 15 plasmas vs. Day 30 plasmas showing UV-resistant subjects (M1; *n *= 9; red) and non-resistant subjects (M2; *n *= 20; green); Supplementary File S6, Figure S4. OPLS-DA Scores plot for Day 15 plasmas (green) vs. Day 30 plasmas (blue) for the UV-resistant subjects (*n *= 9); Supplementary File S6, Figure S5. Univariate analysis (Mann–Whitney) on plasma metabolites for UV resistant subjects (*n *= 9) at Day 15 (D15; following a restricted diet) vs. Day 30 (D30; following two weeks of grape consumption. Reference cited in Supplementary File S5 [22,23,27,28,38,60,61,62,63,64,65,66,67,68,69,70,71,72,73,74,75,76,77,78,79,80,81,82,83,84,85,86,87,88,89,90,91,92].
