# Supplementary material for: Short-Term Grape Consumption Diminishes UV-Induced Skin Erythema
Source: Antioxidants (Basel). 2022 Nov 30;11(12):2372. doi: 10.3390/antiox11122372 (PMC9774720; doi:10.3390/antiox11122372)
Supplement: Supplementary file 1 [file antioxidants-11-02372-s001.zip › antioxidants-2046762-supplementary.pdf]

Supplemental Information

Short-term grape consumption diminishes UV-induced skin erythema

John M. Pezzuto, Asim Dave, Eun-Jung Park, Diren Beyoğlu, Jeffrey R. Idle

Contents

Supplementary File S1. Inclusion/Exclusion Criteria

Supplementary File S2. Subject Responsibilities

Supplementary File S3. Dosing Protocol for Grape Powder

Supplementary File S4. Alpha- and Beta-Diversity

Supplementary File S5. Comparative Taxonomy, Enzyme Levels, and KEGG Orthology

Supplementary File S6. Metabolomic Analyses

Supplementary File S1. Inclusion/Exclusion Criteria

Inclusion Criteria

A subject is considered eligible for participation in the study if all of the following inclusion criteria are satisfied prior to randomization:

1. Is a healthy male or female (confirmed by medical history);
2. Is between the ages of 19 - 55 years of age;
3. Is a non-smoker;
4. Maintained a healthy weight;
5. Consumed little to no alcoholic beverages, or in moderation when consumed;
6. Has not consumed recreational drugs for one week prior;
7. Is willing to abstain from chronic acetaminophen, NSAID and COX-2 inhibitor use for duration of study;
8. Agrees not to participate in any clinical or patch test studies at Day 1 through study completion;
9. In the case of a female of childbearing potential was using two acceptable forms of birth control since last menses (oral/implant/injectable/transdermal contraceptives, intra vaginal ring, intrauterine device (IUD), condom, diaphragm, spermicidal agent, abstinence, partner's vasectomy, tubal ligation). Abstinence or vasectomies were acceptable if the female subject agreed to implement two of the other acceptable methods of birth control if her lifestyle/partner changed;
10. In the case of a female of childbearing potential, has a negative urine pregnancy test (UPT) on Day 1 and is willing to submit to a UPT at the end of study (EOS);
11. In the case of a female of non-childbearing potential: has had a hysterectomy, surgical bilateral oophorectomy and/ or bilateral salpingectomy, or was postmenopausal (at least 1 year with no menses prior to enrollment);
12. Is free of any systemic or dermatological disorder, which, in the opinion of the Investigator, will interfere with the study results or increase the risk of adverse events;
13. Has uniformly-colored skin on the mid to lower back which will allow discernment of erythema, and has Fitzpatrick Skin Types I, II or III;
14. Completes a medical screening procedure; and
15. Reads, understands, and signs an informed consent.

Exclusion Criteria

A subject who has any of the following will be excluded from the study:

1. Has skin on the Fitzpatrick scale IV-VI;
2. Has a history of photosensitivity or photoallergy; melanoma or non-melanoma skin cancers;
3. Has any visible skin disease at the test site which, in the opinion of the Investigator, will interfere with the evaluation of the test site reaction;

4. Has a recent history of vitiligo, melisma and other disorders of pigmentation with the exception of post-inflammatory hyperpigmentation;

5. Is using nasally inhaled/systemic/topical corticosteroids within 4 weeks prior to and/or during the study, or systemic/topical antihistamines 72 hours prior to and during the study;

6. Is using certain antifungal drugs, antihistamines (including diphenhydramine, or Benadryl), antibiotics (including "sulfa" drugs, quinolones and tetracyclines), oral diabetes drugs, sulfonylureas, diuretics, and tricyclic antidepressants. Some herbal supplements such as St. John's wort would also make a person ineligible;

7. Is not willing to refrain from using acetaminophen (occasional use permitted, except within 48 hours of a study visit) or systemic/topical anti-inflammatory analgesics such as aspirin, Aleve, Motrin, Advil, Orudis, or Nuprin for 72 hours prior to and during the study;

8. Is taking medication known to cause photosensitive or phototoxic reactions (e.g., tetracyclines, thiazides, nonsteroidal anti-inflammatory drugs;

9. Any of the following in the 4 weeks prior to start of study:

   a. Major surgery for any indication
   b. Is on cytotoxic chemotherapy for any indication (including methotrexate for arthritis)
   c. Hormonal therapy for cancer prevention (including tamoxifen). Note: treatment with finasteride/dutasteride for BPH did not render a participant ineligible
   d. Topical medications for treatment at the skin site being evaluated (retin A, Accutane, PUVA, 5-FU)
   e. Is taking medication known to cause phototoxic reactions (e.g., tetracyclines, thiazides, nonsteroidal anti-inflammatory drugs [NSAIDS])
   f. Is using medication which, in the opinion of the Investigator, will interfere with the study results (e.g., anti-inflammatory medications, antipsychotics, anticonvulsants with potential pain relief effects, immunomodulatory medications);

10. Is unwilling or unable to refrain from the use of sunscreens, cosmetics, creams, ointments, lotions or similar products on the back during the study;

11. Has psoriasis and/or active atopic dermatitis/eczema;

12. Has a known sensitivity or allergy to constituents of the materials being evaluated;

13. Has nut allergies;

14. Is a female who was pregnant, plans to become pregnant during the study, or is breast feeding a child;

15. Has damaged skin in or around the test sites, including sunburn, excessively deep tans, uneven skin tones, tattoos, scars, excessive hair, numerous freckles, or other disfigurations of the test site;

16. Has received treatment for any type of internal cancer within 5 years prior to study entry;

17. Has a history of or currently being treated for:

a. Hepatitis;
b. Diabetes;
c. Solid organ or bone marrow transplant
d. Keloid formation
e. Chronic renal or hepatic disorder
f. Significant bleeding disorder

18. OTHER

a. Had any condition that might have compromised study results;
b. Is currently or expects to sunbathe or use tanning salons during the study; plans on exposing the irradiated or control areas to the sun
c. Has a history of adverse response (e.g., blistering, sun poisoning) to UV sun lamps/sunlight exposure;
d. Ever had laser resurfacing, dermabrasion, or chemical peel in the test area
e. Is currently participating in any clinical testing;
f. Has any known sensitivity to adhesives, and/or
g. Has received any investigational drug(s) within 28 days from Day 1.

19. Uncontrolled concurrent illness including ongoing or active infection, symptomatic congestive heart failure, unstable angina pectoris, uncontrolled symptomatic cardiac arrhythmia, psychiatric illness/social situations that limited compliance with study requirements or other underlying serious medical condition which, in the investigator's opinion precluded study participation.

Supplementary File S2. Subject Responsibilities

- Skin care products used in the test area that increase sun sensitivity should be excluded during study period including those with alpha-hydroxy acids (AHAs like glycolic acid), beta-hydroxy acids (BHAs like salicylic acid); tretinoins (like Retin A); and benzoyl peroxide.

Subjects should avoid foods that may increase or decrease skin sensitivity.

- Foods that may increase sun sensitivity: celery, dill, fennel, figs, lime, and parsley.

- Foods that may decrease sun sensitivity: citrus, green tea, almonds, red fruits and vegetables, turmeric, olive oil.

Subjects should not consume the following foods during the study:

- Artichokes
- Berries (blueberries, blackberries, grapes, raspberries, strawberries, goji berries, etc.)
- Cocoa
- Dark chocolate
- Pomegranate
- Red wine

Subjects should limit the following foods during the study:

- Coffee/Tea – 1X per day
- Beans/Legumes – 2X per week
- Soy foods (tofu, soy milk, miso, tempeh) 2X per week

Subjects should not consume the following supplements during the study:

- Multivitamin
- Alpha lipoic acid
- B vitamins
- Coenzyme Q10
- Elderberry
- Ellagic acid

- Fish oil
- Flaxseed or flaxseed oil
- Grapeseed extract
- Green tea
- Lycopene
- Niacinamide
- Quercetin
- Resveratrol
- Selenium
- Turmeric
- Vitamin C
- Vitamin E
- Vitamin K

Supplementary File S3. Dosing Protocol for Grape Powder

Important Information:

- Grape powder packets should be stored in a freezer until use.
- Hygroscopic material: protect from water and humidity until reconstituted.
- Drink within 30 minutes of reconstitution.
- Re-stir the grape powder and water just prior to drinking. Please note that the powder doesn't dissolve but creates a suspension.

Purpose: To disperse 36 g of grape powder in 180 ml (6 fl. oz.) of water.

Equipment:

- Clean glass or cup
- Volumetric measuring device
- Filtered or tap water (for reconstitution and rinse)

Procedure:

Step	Instructions
1.	Add approximately 180 ml. (6 fl. oz) of water to cup
2.	Open 36 g packet of grape powder and pour into water in cup.
3.	Stir well, about 30 seconds. If there is any clumping of the powder, break up the clumps by pressing them with the spoon against the glass.
4.	Visually confirm that no un-wetted powder remains. Continue stirring if needed.
5.	Drink within 30 minutes of reconstitution.
6.	Time of Dosing ___________
7.	Rinse container with at least 30 mL (I fl. oz.) of water and drink to ensure getting all of the grape powder.

Supplementary File S4. Alpha- and beta-diversity.

Alpha-diversity

Analysis of alpha-diversity was conducted with the 9 subjects found to demonstrate UV resistance following consumption of grapes for two weeks. Based on OTUs, Chao1 and Shannon analyses, the comparisons revealed no significant differences on the indicated days (Student’s paired *t*-test).

A.

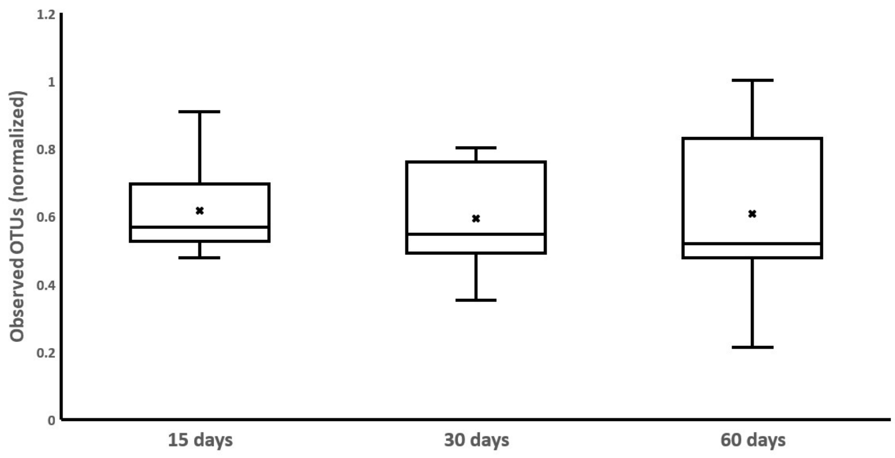

B.

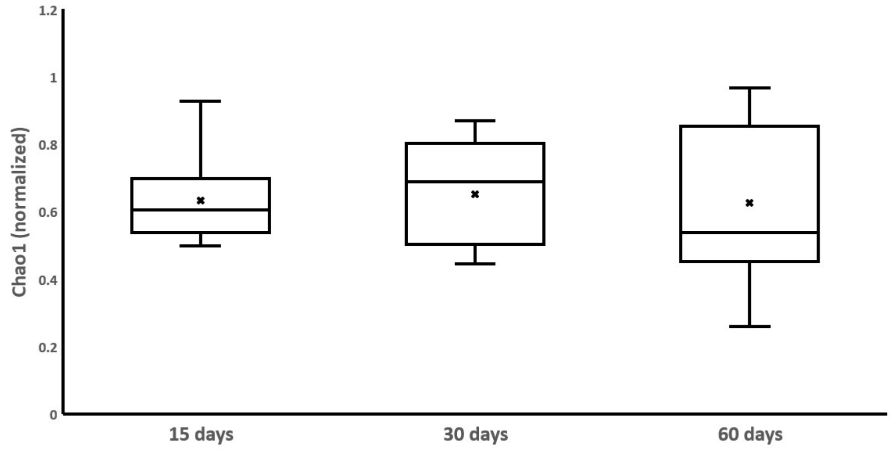

C.

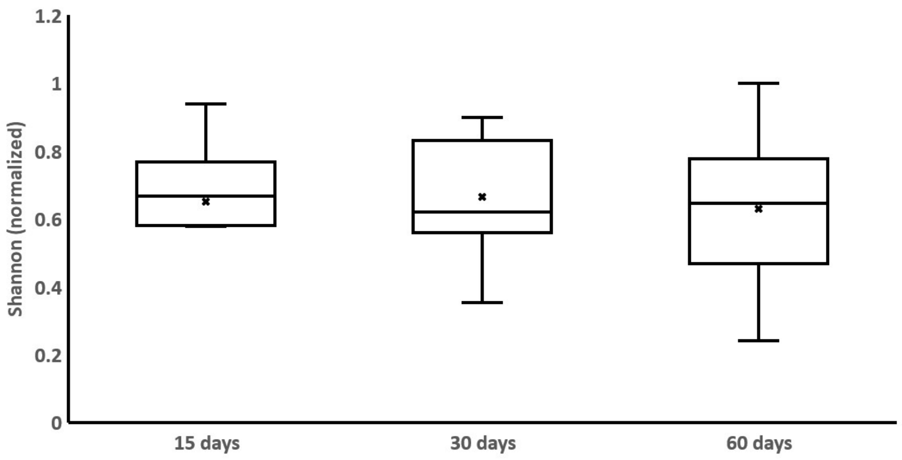

Figure S1. Alpha-diversity. **A.** Observed OTUs. Comparing 15 vs. 30 days: mean 0.616 and 0.593, respectively, $p = 0.60$, Cohen's $d = 0.15$; Comparing 30 vs. 60 days: mean 0.593 and 0.605, respectively, $p = 0.80$, Cohen's $d = 0.05$); Comparing 15 vs. 60 days the mean was 0.616 and 0.605, respectively, $p = 0.86$, Cohen's $d = 0.05$. **B.** Chao1 test. Comparing 15 vs. 30 days: mean 0.631 and 0.651, respectively, $p = 0.70$, Cohen's $d = 0.13$; Comparing 30 vs. 60 days: mean 0.651 and 0.624, respectively, $p = 0.70$, Cohen's $d = 0.13$); Comparing 15 vs. 60 days the mean was 0.631 and 0.624, respectively, $p = 0.89$, Cohen's $d = 0.03$. **C.** Shannon test. Comparing 15 vs. 30 days: mean 0.651 and 0.663, respectively, $p = 0.88$, Cohen's $d = 0.06$; Comparing 30 vs. 60 days: mean 0.663 and 0.630, respectively, $p = 0.68$, Cohen's $d = 0.16$); Comparing 15 vs. 60 days the mean was 0.651 and 0.630, respectively, $p = 0.65$, Cohen's $d = 0.09$.

Beta-diversity

A.

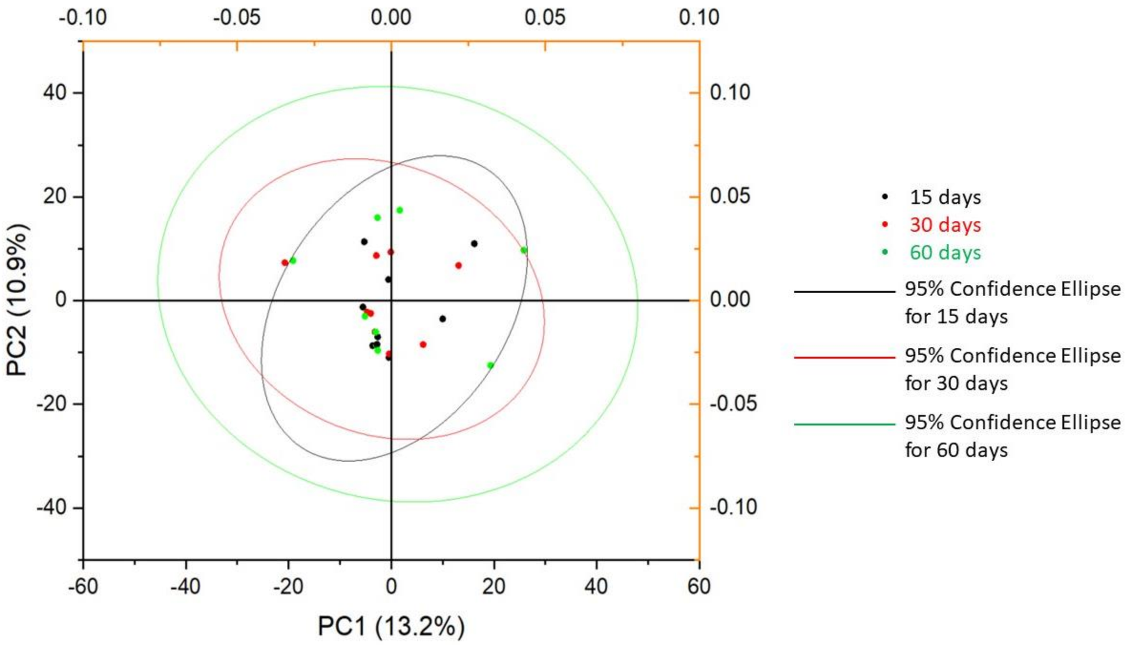

B.

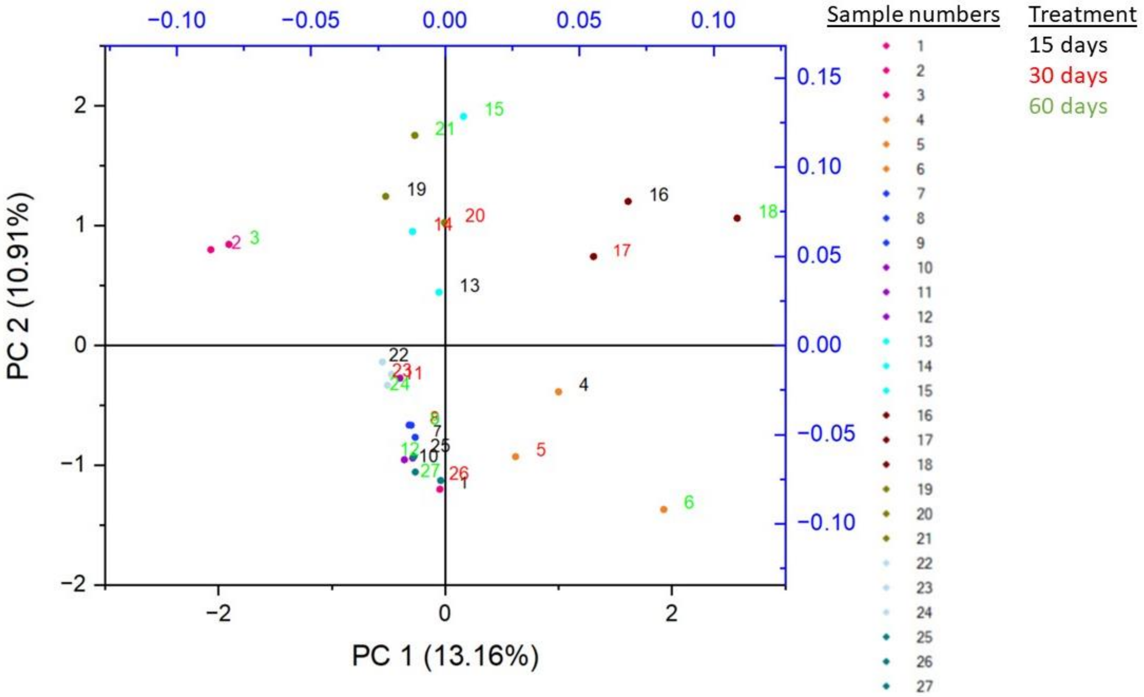

C.

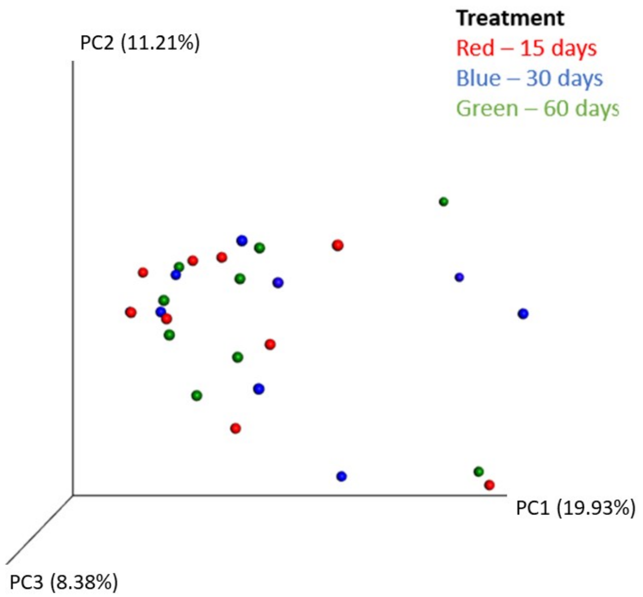

D.

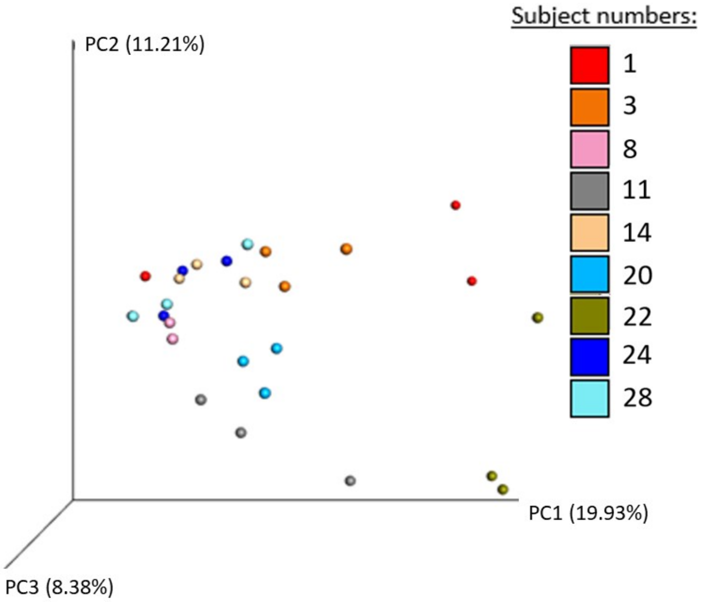

Figure S2. Analysis of beta-diversity was conducted with the 9 subjects found to demonstrate UV resistance following consumption of grapes for two weeks. Cluster analysis (PCA and PCoA plots) revealed no significant differences on Day 15, 30 or 60. **A.** PCA plot and Cluster analysis showing the results on Day 15 (black), Day 30 (red), and Day 60 (green). There were no apparent differences observed in the clusters formed. **B.** PCA plot showing the position of each of the 9 individuals on Day 15, 30 and 60. Each individual is assigned three consecutive numbers (e.g., 1, 2, 3 is individual one) and a single color that can be tracked on the plot. The numbers

within the plot (black, red or green) indicate the position of the individual on the day specified by the color. **C.** PCoA plot for results observed on Day 15 (red), 30 (blue) or 60 (green). **D.** PCoA plot for each subject. The dots denote the samples collected (the same color dots represent one subject).

Supplementary File S5. Comparative Taxonomy, Enzyme Levels, and KEGG Orthology

Table S1. Comparative enzyme levels determined by comparison of Day 15 vs. Day 30 for the subjects resistant to UV irradiation on Day 30 (*n* = 9).

Enzymes	Log2(Fold-change)	*Q* value	*D* value
5.4.3.5 oraS; D-ornithine 4,5-aminomutase subunit alpha	2.121	0.021	1.036
5.3.3.14 fabM; *trans*-2-decenoyl-[acyl-carrier protein] isomerase	1.443	0.098	0.875
2.7.13.3 phoQ; two-component system, OmpR family, sensor histidine kinase PhoQ	3.738	0.10	0.898
1.4.99.5 hcnC; hydrogen cyanide synthase HcnC	-0.778	0.10	0.831
6.3.2.9 murD; UDP-*N*-acetylmuramoylalanine-D-glutamate ligase	-0.117	0.11	0.854
1.18.1.1 rubB, alkT; rubredoxin---NAD^+ reductase	-2.974	0.12	0.905
4.1.1.85 ulaD, sgaH, sgbH; 3-dehydro-L-gulonate-6-phosphate decarboxylase	3.044	0.12	0.912
2.8.3.16 frc; formyl-CoA transferase	2.445	0.13	0.916
1.16.1.9 yqjH; ferric-chelate reductase (NADPH)	3.079	0.14	0.911
6.3.4.13 purD; phosphoribosylamine-glycine ligase	-0.092	0.14	0.936
3.6.3.33 ABC.VB12.A, btuD; vitamin B12 transport system ATP-binding protein	3.953	0.15	0.935
3.5.2.18 ena; enamidase	-3.595	0.16	0.996
3.2.1.89 E3.2.1.89; arabinogalactan endo-1,4-beta-galactosidase	0.466	0.18	0.939
4.1.1.19 pdaD; arginine decarboxylase	1.551	0.19	1.008
2.7.1.55 alsK; allose kinase	1.678	0.28	1.058
3.4.21.62 aprE; subtilisin	2.946	0.38	1.076

Table S2. KEGG pathways altered when comparing Day 15 vs. 30 with the group resistant to UV irradiation on Day 60 (*n* = 9).

Pathways	Log2(Fold-change)	*Q* value	*D* value
1. Oxidoreductases; 1.4 Acting on the CH-NH2 group of donors; 1.4.99 With other acceptors	-0.717	0.098	0.837
Not specified; Hydrogen cyanide production	-0.728	0.13	0.843
1. Oxidoreductases; 1.16 Oxidizing metal ions; 1.16.1 With NAD^+ or $NADP^+$ as acceptor	2.776	0.17	0.901
ABC Transporters, Prokaryotic Type; Phosphate and amino acid transporters; Arginine transporter [MD:M00229]	1.003	0.26	0.934

Table S3. Taxonomic comparison on Day 30 vs. 60 days for the group resistant to UV irradiation on Day 30 (*n* = 9).

Taxonomy	Log2(Fold-change)	*Q* value	*D* value	Functional Connotations
s__*Streptococcus_thermophilus*[1]	-2.757	0.042	1.332	*Streptococcus thermophilus* is widely used as a starter culture in cheese, yogurt, and other cultured dairy products [1].
g__*Holdemania*	2.168	0.083	0.931	Leads to reduction in the vegetarian diet [2].

[1]Taxonomic hierarchies are designated as c (class), o (order), f (family), g (genus) or s (species).

Table S4. Comparative enzyme levels determined by comparison of Day 30 vs. Day 60 for the subjects resistant to UV irradiation on Day 30 (n = 9).

Enzymes	Log2(Fold-change)	*Q* value	*D* value
3.5.1.115 mca; mycothiol *S*-conjugate amidase	-3.288	0.085	1.062
3.1.3.16 pphA; serine/threonine protein phosphatase 1	-1.340	0.087	1.074
5.3.1.6 rpiB; ribose 5-phosphate isomerase B	0.238	0.096	1.030
2.7.13.3 dcuS; two-component system, CitB family, sensor histidine kinase DcuS	-0.630	0.10	0.835
1.1.1.159 hdhA; 7-alpha-hydroxysteroid dehydrogenase	-1.173	0.10	0.839
5.3.1.23 mtnA; methylthioribose-1-phosphate isomerase	-0.627	0.10	1.062
4.2.1.51 pheA; chorismate mutase / prephenate dehydratase	0.178	0.11	0.842
5.4.99.5 pheA; chorismate mutase / prephenate dehydratase	0.178	0.11	0.842
3.5.4.19 hisI; phosphoribosyl-AMP cyclohydrolase	0.128	0.12	0.908
3.5.3.12 aguA; agmatine deiminase	0.347	0.12	0.874
2.8.3.17 fldA; cinnamoyl-CoA:phenyllactate CoA-transferase	0.971	0.12	0.868
1.8.4.8 cysH; phosphoadenosine phosphosulfate reductase	-1.186	0.12	1.079
1.8.4.10 cysH; phosphoadenosine phosphosulfate reductase	-1.186	0.14	1.079
3.5.4.21 codA; cytosine/creatinine deaminase	-1.643	0.17	1.121
5.3.1.24 trpF; phosphoribosylanthranilate isomerase	0.185	0.23	1.154
3.5.4.1 codA; cytosine/creatinine deaminase	-1.643	0.23	1.121
4.2.1.109 mtnB; methylthioribulose-1-phosphate dehydratase	-0.902	0.34	1.277

Table S5. KEGG pathways altered when comparing Day 30 vs. 60 with the group resistant to UV irradiation on Day 60 (n = 9).

Pathways	Log2(Fold-change)	*Q* value	*D* value
Extracellular nucleation-precipitation pathway; Curli assembly protein	-13.392	0.11	435.398
Heat shock proteins; HSP90	-0.688	0.14	4.068

Major Facilitator Superfamily (MFS); Organic acid transporters; Sialate:H^+ symporter (SHS) family [TC:2.A.1.12]	-6.737	0.15	3.868
1. Oxidoreductases; 1.3 Acting on the CH-CH group of donors; 1.3.3 With oxygen as acceptor	-12.814	0.27	501.011

Table S6. Taxonomic comparison on Day 15 vs. Day 60 for the group resistant to UV irradiation on Day 30 (*n* = 9).

Taxonomy	Log2(Fold-change)	*Q* value	*D* value	Functional Connotations
s__*Blautia_wexlerae*[1]	0.751	0.079	0.970	Depletion is associated with insulin resistance in obese individuals [3].
s__*Collinsella_aerofaciens*	1.448	0.089	0.891	An altered abundance of *C. aerofaciens* may be linked with several health disorders, including irritable bowel syndrome [4].
g__*Faecalicatena*	0.802	0.095	0.973	Levels of *Faecalicatena lactaris* associated with ABO, and suggested preferential utilization of secreted blood antigens as energy source in the gut [5].
g__*Catonella*	-2.456	0.11	1.098	Was described as a putative periodontal pathogen, as it was more abundantly found in chronic periodontitis subjects [6].
f__*Lachnospiraceae*	0.878	0.16	1.159	Members of this family have been linked to obesity and protection from colon cancer in humans, mainly due to the association of many species within the group with the production of butyric acid, a substance that is important for both microbial and host epithelial cell growth [7].

[1]Taxonomic hierarchies are designated as c (class), o (order), f (family), g (genus) or s (species).

Table S7. Comparative enzyme levels determined by comparison of Day 15 vs. Day 60 for the subjects resistant to UV irradiation on Day 30 (n = 9).

Enzymes	Log2(Fold-change)	*Q* value	*D* value
3.5.1.115 mca; mycothiol *S*-conjugate amidase	-3.288	0.085	1.062
3.1.3.16 pphA; serine/threonine protein phosphatase 1	-1.340	0.089	1.074
5.3.1.6 rpiB; ribose 5-phosphate isomerase B	0.238	0.095	1.030
5.4.99.5 pheA; chorismate mutase / prephenate dehydratase	0.178	0.10	0.842
2.7.13.3 dcuS; two-component system, CitB family, sensor histidine kinase DcuS	-0.630	0.10	0.835
1.1.1.159 hdhA; 7-alpha-hydroxysteroid dehydrogenase	-1.173	0.10	0.839
5.3.1.23 mtnA; methylthioribose-1-phosphate isomerase	-0.627	0.10	1.062
4.2.1.51 pheA; chorismate mutase / prephenate dehydratase	0.178	0.11	0.842
3.5.4.19 hisI; phosphoribosyl-AMP cyclohydrolase	0.128	0.12	0.908
3.5.3.12 aguA; agmatine deiminase	0.347	0.12	0.874
2.8.3.17 fldA; cinnamoyl-CoA:phenyllactate CoA-transferase	0.971	0.12	0.868
1.8.4.8 cysH; phosphoadenosine phosphosulfate reductase	-1.186	0.12	1.079
1.8.4.10 cysH; phosphoadenosine phosphosulfate reductase	-1.186	0.14	1.079
3.5.4.21 codA; cytosine/creatinine deaminase	-1.643	0.17	1.121
5.3.1.24 trpF; phosphoribosylanthranilate isomerase	0.185	0.23	1.154
3.5.4.1 codA; cytosine/creatinine deaminase	-1.643	0.23	1.121
4.2.1.109 mtnB; methylthioribulose-1-phosphate dehydratase	-0.902	0.34	1.277

Table S8. KEGG pathways altered when comparing Day 15 vs. 60 with the group resistant to UV irradiation on Day 60 (n = 9).

Pathways	Log2(Fold-change)	*Q* value	*D* value
Metallo Peptidases; Family M24	9.998	0.11	8.916
ABC Transporters, Prokaryotic Type; ABC-2 type and other transporters;	8.104	0.11	3.615

Lipopolysaccharide transporter [MD:M00320]			
1. Oxidoreductases; 1.3 Acting on the CH-CH group of donors; 1.3.3 With oxygen as acceptor	-11.852	0.14	501.385
CitB family; DcuS-DcuR	0.960	0.21	0.682
3. Hydrolases; 3.2 Glycosylases; 3.2.- Glycosylases	7.857	0.29	9.131

Table S9. Taxonomic comparison of the group resistant to UV irradiation on Day 60 (*n* = 9) with the group of non-responsive subjects (*n* = 20) on Day 15.

**Taxonomy**	**Log2(Fold-change)**	***Q* value**	**Functional Connotations**
f__*Prevotellaceae*[1]	-2.124	0.077	Participate in human disease by promoting chronic inflammation [8].
g__*Emergencia*	1.501	0.10	Basis for anaerobic TMA generation from the L-carnitine–derived metabolite γ-butyrobetaine (γbb) [9].
g__*Citrobacter*	-6.221	0.12	Higher frequency in Irritable bowel syndrome [10].
g__*Catonella*	-3.697	0.13	Elevates the risk of developing gastric cancer [11].
s__*Prevotella_copri*	-3.083	0.14	Participate in human disease by promoting chronic inflammation [8].
g__*Prevotella*	-3.002	0.28	Participate in human disease by promoting chronic inflammation [8].

[1]Taxonomic hierarchies are designated as c (class), o (order), f (family), g (genus) or s (species).

Table S10. Comparative enzyme levels determined by comparing group resistant to UV irradiation on Day 60 (*n* = 9) with the group of non-responsive subjects (*n* = 20) on Day 15.

Enzymes	Log2(Fold-change)	*Q* value
2.3.1.118 nhoA; *N*-hydroxyarylamine *O*-acetyltransferase	-3.808	0.00
2.6.1.92 pseC; UDP-4-amino-4,6-dideoxy-L-*N*-acetyl-beta-L-altrosamine transaminase	-3.120	0.017
3.5.4.21 codA; cytosine/creatinine deaminase	-2.405	0.019
3.5.4.1 codA; cytosine/creatinine deaminase	-2.405	0.021
5.1.99.8 folB; 7,8-dihydroneopterin aldolase/epimerase/oxygenase	-2.294	0.025
4.1.2.25 folB; 7,8-dihydroneopterin aldolase/epimerase/oxygenase	-2.294	0.026
6.3.2.14 entE, dhbE, vibE, mxcE; 2,3-dihydroxybenzoate-AMP ligase	-2.336	0.027
7.1.1.2 nuoC; NADH-quinone oxidoreductase subunit C	2.518	0.027
2.7.7.59 glnD; [protein-PII] uridylyltransferase	-2.809	0.027
3.1.21.7 E3.1.21.7, nfi; deoxyribonuclease V	-4.077	0.027
1.13.11.81 folB; 7,8-dihydroneopterin aldolase/epimerase/oxygenase	-2.294	0.027
6.3.2.7 murE; UDP-*N*-acetylmuramoyl-L-alanyl-D-glutamate-L-lysine ligase	-1.577	0.028
3.5.2.18 ena; enamidase	-2.694	0.029
1.18.1.1 rubB, alkT; rubredoxin---NAD^+ reductase	-3.210	0.030
3.1.13.5 rnd; ribonuclease D	-1.960	0.030
2.7.7.61 citX; holo-ACP synthase	-1.532	0.030
1.17.5.3 fdnG; formate dehydrogenase-*N*, alpha subunit	-2.047	0.030
3.2.1.10 IMA, malL; oligo-1,6-glucosidase	-1.928	0.030
1.3.7.7 chlB; light-independent protochlorophyllide reductase subunit B	-1.019	0.030

2.4.1.25 malQ; 4-alpha-glucanotransferase	-1.504	0.030
2.1.1.174 rlmG; 23S rRNA (guanine1835-N2)-methyltransferase	-2.589	0.031
2.4.1.7 E2.4.1.7; sucrose phosphorylase	-2.131	0.031
3.4.23.43 pilD, pppA; leader peptidase (prepilin peptidase) / *N*-methyltransferase	-1.785	0.032
2.7.13.3 dpiB, citA; two-component system, CitB family, cit operon sensor histidine kinase CitA	-1.452	0.032
3.6.4.13 rhlB; ATP-dependent RNA helicase RhlB	-1.275	0.035
4.2.1.109 mtnB; methylthioribulose-1-phosphate dehydratase	-1.012	0.039
2.7.7.58 entE, dhbE, vibE, mxcE; 2,3-dihydroxybenzoate-AMP ligase	-2.336	0.041

Table S11. KEGG pathways altered (KO level 3) when comparing group resistant to UV irradiation on Day 60 (*n* = 9) with the group of non-responsive subjects (*n* = 20) on Day 15.

**Pathways**	**Log2(Fold-change)**	***Q* value**
Nonribosomal peptide synthetase (NRPS); Nonlinear NRPS; Vibriobactin synthetase	-2.224	0.021
Nonribosomal peptide synthetase (NRPS); Iterative NRPS; Bacillibactin synthetase	-2.153	0.021
Nonribosomal peptide synthetase (NRPS); Iterative NRPS; Enterobactin synthetase	-2.066	0.021
Aspartic Peptidases; Family A24: type IV prepilin peptidase family	-1.906	0.028
Major Facilitator Superfamily (MFS); Organic acid transporters; Metabolite:H^+ symporter (MHS) family [TC:2.A.1.6]	-1.753	0.029
3. Hydrolases; 3.1 Acting on ester bonds; 3.1.13 Exoribonucleases producing 5-phosphomonoesters	-1.459	0.029
Type II toxins: Membrane damaging toxins; Pore-forming toxins, RTX	-1.939	0.029
Prokaryotic Type; 3processing and CCA adding factors; 3processing factor	-0.268	0.030

CitB family; CitA-CitB	-1.470	0.030
3. Hydrolases; 3.4 Acting on peptide bonds (peptidases); 3.4.17 Metallocarboxypeptidases	0.549	0.030
Histidine protein kinases; CitB family	-0.742	0.031
Prokaryotic Type; Helix-turn-helix; TetR/AcrR family	-0.590	0.032
Nonribosomal peptide synthetase (NRPS); Nonlinear NRPS; Myxochelin synthetase	-2.224	0.032
Phosphotransferase System (PTS); Enzyme I and HPr; Phosphocarrier protein HPr [TC:8.A.8 4.A.2]	-0.902	0.033
1. Oxidoreductases; 1.15 Acting on superoxide as acceptor; 1.15.1 Acting on superoxide as acceptor (only sub-subclass identified to date)	0.326	0.034
1. Oxidoreductases; 1.6 Acting on NADH or NADPH; 1.6.1 With NAD^+ or $NADP^+$ as acceptor	-2.747	0.034
5. Isomerases; 5.1 Racemases and epimerases; 5.1.2 Acting on hydroxy acids and derivatives	-3.352	0.034
Metallo Peptidases; Family M24	0.120	0.034
Serine Peptidases; Family S9: prolyl oligopeptidase family	-2.868	0.035
Major Facilitator Superfamily (MFS); Sugar transporters; Oligosaccharide:H^+ symporter (OHS) family [TC:2.A.1.5]	-2.137	0.035

Table S12. Taxonomic comparison of the group resistant to UV irradiation on Day 60 (*n* = 9) with the group of non-responsive subjects (*n* = 20) on Day 60.

**Taxonomy**	**Log2(Fold-change)**	***Q* value**	**Functional Connotations**
s__*Roseburia_faecis*[1]	-2.245	0.012	Metabolize dietary components that stimulate their proliferation and metabolic activities [12].
g__*Roseburia*	-1.282	0.048	Metabolize dietary components that stimulate their proliferation and metabolic activities [12].
s__*Ruminococcus_callidus*	-3.870	0.050	Enriched in rheumatoid arthritis patient [13].

g__*Erysipelatoclostridium*	-0.825	0.051	Anaerobic enteric bacterium that is part of the normal human gut microbiota [14].
g__*Intestinibacter*	-1.745	0.055	Butyrate-producing bacteria [15].
g__*Fusicatenibacter*	-0.741	0.059	Decreased in the case of ulcerative colitis [16].
s__*Fusicatenibacter_saccharivorans*	-0.742	0.064	Decreased in the case of ulcerative colitis [16].
g__*Anaerostipes*	-0.992	0.069	A butyrate producer [17].
g__*Ileibacterium*	-1.413	0.076	Butyrate-producing bacteria [18].
g__*Clostridium*	-0.980	0.084	An increased abundance of *Clostridiaceae* was shared by both IBD-A and rheumatoid arthritis patients [19].
f__*Lachnospiraceae*	-0.578	0.086	Main producers of short-chain fatty acids [20].
f__*Clostridiaceae*	-0.955	0.096	An increased abundance of *Clostridiaceae* was shared by both IBD-A and rheumatoid arthritis patients [19].

[1]Taxonomic hierarchies are designated as c (class), o (order), f (family), g (genus) or s (species).

Table S13. Comparative enzyme levels determined by comparing group resistant to UV irradiation on Day 60 (n = 9) with the group of non-responsive subjects (n = 20) on Day 60.

**Enzymes**	**Log2(Fold-change)**	***Q* value**
2.7.13.3 dpiB, citA; two-component system, CitB family, cit operon sensor histidine kinase CitA	-1.596	0.042
6.3.2.14 entE, dhbE, vibE, mxcE; 2,3-dihydroxybenzoate-AMP ligase	-2.330	0.044
3.4.19.11 yqgT; g-D-glutamyl-meso-diaminopimelate peptidase	-1.336	0.048
2.7.13.3 prrB; two-component system, OmpR family, sensor histidine kinase PrrB	-1.724	0.048
1.4.1.2 gudB, rocG; glutamate dehydrogenase	-2.736	0.053
3.5.1.5 ureAB; urease subunit gamma/beta	-1.400	0.055
1.1.1.251 E1.1.1.251, gatD; galactitol-1-phosphate 5-dehydrogenase	-1.050	0.057

3.2.1.176 celS; cellulose 1,4-beta-cellobiosidase	-5.187	0.058
2.7.7.58 entE, dhbE, vibE, mxcE; 2,3-dihydroxybenzoate-AMP ligase	-2.330	0.059
3.1.1.24 pcaD; 3-oxoadipate enol-lactonase	-1.044	0.060
2.3.3.14 LYS21, LYS20; homocitrate synthase	-3.341	0.060
1.1.1.281 rmd; GDP-4-dehydro-6-deoxy-D-mannose reductase	-1.162	0.061
1.17.2.1 nicA; nicotinate dehydrogenase subunit A	-3.213	0.062
2.7.1.51 fucK; L-fuculokinase	-1.256	0.064
2.4.1.280 chbP; *N*,*N*'-diacetylchitobiose phosphorylase	-1.047	0.064
3.5.4.25 ribA, RIB1; GTP cyclohydrolase II	-1.120	0.066

Table S14. KEGG pathways altered (KO level 3) when comparing group resistant to UV irradiation on Day 60 (*n* = 9) with the group of non-responsive subjects (*n* = 20) on Day 60.

**Pathways**	**Log2(Fold-change)**	***Q* value**
Nonribosomal peptide synthetase (NRPS); Nonlinear NRPS; Vibriobactin synthetase	-2.200	0.029
Nonribosomal peptide synthetase (NRPS); Nonlinear NRPS; Myxochelin synthetase	-2.200	0.033
Nonribosomal peptide synthetase (NRPS); Iterative NRPS; Bacillibactin synthetase	-2.137	0.038
Nonribosomal peptide synthetase (NRPS); Nonlinear NRPS; Mycobactin synthetase	-3.342	0.038
Aspartic Peptidases; Family A25: gpr protease family	-0.475	0.038
CitB family; CitA-CitB	-1.600	0.040
OmpR family; CssS-CssR	-0.496	0.040

Metallo Peptidases; Family M14: carboxypeptidase A family	-1.279	0.047
ABC Transporters, Prokaryotic Type; Oligosaccharide and polyol transporters; Trehalose/maltose transporter [MD:M00204]	-1.240	0.060
ABC Transporters, Prokaryotic Type; Monosaccharide transporters; Putative simple sugar transporter [MD:M00221]	-0.552	0.070

Table S15. Comparative enzyme levels determined when comparing group resistant to UV irradiation on Day 30 and Day 60 (*n* = 3) with the group of non-responsive subjects (*n* = 20) on Day 15.

Enzymes	Log2(Fold-change)	*Q* value
7.1.1.2 nuoC; NADH-quinone oxidoreductase subunit C	2.866	0.00
1.1.1.132 algD; GDP-mannose 6-dehydrogenase	1.747	0.00
2.7.13.3 comP; two-component system, NarL family, sensor histidine kinase ComP	1.027	0.00
2.7.2.4 metL; bifunctional aspartokinase / homoserine dehydrogenase 2	-5.910	0.036
4.2.1.12 edd; phosphogluconate dehydratase	-5.259	0.036
4.2.1.99 acnB; aconitate hydratase 2 / 2-methylisocitrate dehydratase	-5.205	0.037
2.7.3.9 PTS-EI.PTSP, ptsP; phosphotransferase system, enzyme I, PtsP	-5.539	0.037
1.1.1.3 metL; bifunctional aspartokinase / homoserine dehydrogenase 2	-5.910	0.038
4.2.1.3 acnB; aconitate hydratase 2 / 2-methylisocitrate dehydratase	-5.205	0.040
1.8.99.5 dsrA; dissimilatory sulfite reductase alpha subunit	0.745	0.041
4.6.1.1 cyaA; adenylate cyclase, class 1	-5.227	0.042
2.3.1.15 plsB; glycerol-3-phosphate *O*-acyltransferase	-5.981	0.046
1.7.2.3 torA; trimethylamine-*N*-oxide reductase (cytochrome c)	-5.297	0.046
1.1.1.403 dthD; D-threitol dehydrogenase (NAD^+)	-5.859	0.046
3.6.3.50 fliI; flagellum-specific ATP synthase	-5.146	0.047
2.1.1.266 rlmJ; 23S rRNA (adenine2030-N6)-methyltransferase	-4.572	0.048
5.3.3.8 fadB; 3-hydroxyacyl-CoA dehydrogenase / enoyl-CoA hydratase / 3-hydroxybutyryl-CoA epimerase / enoyl-CoA isomerase	-5.939	0.050
3.2.1.179 ugl; gellan tetrasaccharide unsaturated glucuronyl hydrolase	0.769	0.050
4.1.2.21 dgoA; 2-dehydro-3-deoxyphosphogalactonate aldolase	-5.181	0.055
5.1.2.3 fadB; 3-hydroxyacyl-CoA dehydrogenase / enoyl-CoA hydratase / 3-hydroxybutyryl-CoA epimerase / enoyl-CoA isomerase	-5.939	0.056
4.2.1.17 fadB; 3-hydroxyacyl-CoA dehydrogenase / enoyl-CoA hydratase / 3-hydroxybutyryl-CoA epimerase / enoyl-CoA isomerase	-5.939	0.063
1.1.1.35 fadB; 3-hydroxyacyl-CoA dehydrogenase / enoyl-CoA hydratase / 3-hydroxybutyryl-CoA epimerase / enoyl-CoA isomerase	-5.939	0.072
1.1.1.339 tll; dTDP-6-deoxy-*L*-talose 4-dehydrogenase (NAD^+)	1.757	0.073

Table S16. KEGG pathways altered (KO level 3) when comparing group resistant to UV irradiation on Day 30 and Day 60 (n = 3) with the group of non-responsive subjects (n = 20) on Day 15.

Pathway	Log2(Fold-change)	*Q* value
3. Hydrolases; 3.2 Glycosylases; 3.2.2 Hydrolysing *N*-glycosyl compounds	-4.186	0.00
ABC Transporters, Prokaryotic Type; Metallic cation, iron-siderophore and vitamin B12 transporters; Zinc transporter [MD:M00242]	-4.463	0.00
Aspartic Peptidases; Family A24: type IV prepilin peptidase family	-4.082	0.00
Nonribosomal peptide synthetase (NRPS); Nonlinear NRPS; Mycobactin synthetase	-3.573	0.0016
Nonribosomal peptide synthetase (NRPS); Iterative NRPS; Enterobactin synthetase	-4.010	0.0018
Nonribosomal peptide synthetase (NRPS); Nonlinear NRPS; Vibriobactin synthetase	-3.477	0.0022
Nonribosomal peptide synthetase (NRPS); Nonlinear NRPS; Myxochelin synthetase	-3.477	0.0027
Major Facilitator Superfamily (MFS); Sugar transporters; Oligosaccharide:H^+ symporter (OHS) family [TC:2.A.1.5]	-4.535	0.0098
ABC Transporters, Prokaryotic Type; Metallic cation, iron-siderophore and vitamin B12 transporters; Iron/zinc/copper transporter [MD:M00318]	-4.403	0.0099
Cysteine Peptidases; Family C11: clostripain family	-3.655	0.011
Major Facilitator Superfamily (MFS); Organic acid transporters; Phenylproprionate permease (PPP) [TC:2.A.1.27]	-3.574	0.012

Table S17. Taxonomic comparison of the group resistant to UV irradiation on Day 30 and Day 60 (n = 3) with the group of non-responsive subjects (n = 20) on Day 30.

Taxonomy	Log2(Fold-change)	*Q* value	Functional Connotations
g__*Peptostreptococcus*[1]	-2.792	0.036	Identified from clinical samples of diseased individuals [21].
s__*Blautia*_sp._KLE_1732	-3.132	0.046	Probiotic properties [22].
s__*Collinsella_stercoris*	-4.131	0.049	Abundance of the genus *Collinsella* is positively

			correlated with circulating insulin [23].
s__*Ruminococcus_bicirculans*	-9.103	0.051	Enriched in rheumatoid arthritis patient [13].
g__*Lactobacillus*	-3.330	0.055	Probiotic properties [24].
f__*Lactobacillaceae*	-3.313	0.063	Probiotic properties [24].

[1]Taxonomic hierarchies are designated as c (class), o (order), f (family), g (genus) or s (species).

Table S18. Comparative enzyme levels determined when comparing group resistant to UV irradiation on Day 30 and Day 60 (n = 3) with the group of non-responsive subjects (n = 20) on Day 30.

Enzymes	Log2(Fold-change)	*Q* value
3.2.1.10 IMA, malL; oligo-1,6-glucosidase	-3.612	0.00
1.2.1.8 betB, gbsA; betaine-aldehyde dehydrogenase	-4.422	0.040
1.4.1.2 gudB, rocG; glutamate dehydrogenase	-3.426	0.040
2.7.7.7 holC; DNA polymerase III subunit chi	-3.291	0.042
1.17.1.1 ascD, ddhD, rfbI; CDP-4-dehydro-6-deoxyglucose reductase, E3	-3.680	0.042
2.3.2.3 mprF, fmtC; phosphatidylglycerol lysyltransferase	-3.615	0.044
4.1.3.38 pabC; 4-amino-4-deoxychorismate lyase	-4.039	0.045
1.7.1.6 azr; azobenzene reductase	-3.444	0.047
3.1.1.27 pdla; 4-pyridoxolactonase	-4.549	0.047
2.7.1.177 pduX; L-threonine kinase	-3.130	0.050
6.6.1.2 cobS; cobaltochelatase CobS	-3.259	0.051
3.2.1.70 dexB; glucan 1,6-alpha-glucosidase	-4.104	0.051
2.1.1.174 rlmG; 23S rRNA (guanine1835-*N*2)-methyltransferase	-3.577	0.053
6.2.1.14 bioW; 6-carboxyhexanoate--CoA ligase	-3.533	0.053
1.2.7.10 oorB; oxalate oxidoreductase subunit beta	-3.088	0.054
6.3.2.14 entE, dhbE, vibE, mxcE; 2,3-dihydroxybenzoate-AMP ligase	-4.187	0.068

Table S19. KEGG pathways altered (KO level 3) when comparing group resistant to UV irradiation on Day 30 and Day 60 (n = 3) with the group of non-responsive subjects (n = 20) on Day 30.

Pathway	Log2(Fold-change)	*Q* value
OmpR family; BaeS-BaeR	-1.180	0.0045
ABC Transporters, Prokaryotic Type; Oligosaccharide and polyol transporters; Putative *sn*-Glycerol 3-phosphate transporter [MD:M00198]	-1.101	0.0090
Histidine protein kinases; CitB family	-1.450	0.011
Metallo Peptidases; Family M38: beta-aspartyl dipeptidase family	-1.087	0.012
ABC Transporters, Prokaryotic Type; ABC-2 type and other transporters; Teichoic acid transporter [MD:M00251]	-0.867	0.012
Nonribosomal peptide synthetase (NRPS); Nonlinear NRPS; Mycobactin synthetase	-5.080	0.012
CitB family; CitA-CitB	-2.067	0.012
Nonribosomal peptide synthetase (NRPS); Iterative NRPS; Bacillibactin synthetase	-4.896	0.012
3. Hydrolases; 3.3 Acting on ether bonds; 3.3.1 Thioether and trialkylsulfonium hydrolases	-1.198	0.013

Table S20. Taxonomic comparison of the group resistant to UV irradiation on Day 30 and Day 60 (*n* = 3) with the group of non-responsive subjects (*n* = 20) on Day 60.

Taxonomy	Log2(Fold-change)	*Q* value	Functional Connotations
s__*Roseburia_faecis*[1]	-2.432	0.0070	Produces butyrate in the colon [25].
g__*Gemella*	2.049	0.010	Found to be higher in oral squamous cell carcinoma (OSCC) [26].
s__*Bacteroides_ovatus*	-1.471	0.014	Harbor an extensive repertoire of enzymes for carbohydrate Fermentation, which generates volatile fatty acids and other metabolites [27].
g__*Paraprevotella*	-2.936	0.021	Potential effects on human health are unknown [28].
s__*Ruminococcus_bicirculans*	-9.556	0.023	Enriched in rheumatoid arthritis patient [13].
s__*Paraprevotella_clara*	-2.948	0.025	Potential effects on human health are unknown [28].
s__*Clostridium_sp._AT4*	-5.445	0.026	An increased abundance of Clostridiaceae was shared by both IBD and RA patients [19].

[1]Taxonomic hierarchies are designated as c (class), o (order), f (family), g (genus) or s (species).

Table S21. Comparative enzyme levels determined when comparing group resistant to UV irradiation on Day 30 and Day 60 (*n* = 3) with the group of non-responsive subjects (*n* = 20) on Day 60.

Enzymes	Log2(Fold-change)	*Q* value
6.6.1.2 cobS; cobaltochelatase CobS	-3.168	0.040
3.5.3.1 E3.5.3.1, rocF, arg; arginase	2.190	0.042
3.1.1.17 E3.1.1.17, gnl, RGN; gluconolactonase	2.225	0.047
1.6.1.2 pntA; H^+-translocating NAD(P) transhydrogenase subunit alpha	-3.386	0.049
2.7.1.163 hph; hygromycin-B 4-*O*-kinase	-4.267	0.051
2.4.1.317 tylN; *O*-mycaminosyltylonolide 6-deoxyallosyltransferase	2.225	0.051
4.2.1.22 CBS; cystathionine beta-synthase	-4.684	0.051
1.17.1.1 ascD, ddhD, rfbI; CDP-4-dehydro-6-deoxyglucose reductase, E3	-5.298	0.051
2.7.1.12 E2.7.1.12, gntK, idnK; gluconokinase	-4.816	0.053
2.4.1.301 kanE, kanM2; 2'-deamino-2'-hydroxyneamine 1-alpha-D-kanosaminyltransferase	0.986	0.053

7.1.1.1 pntB; H^+-translocating NAD(P) transhydrogenase subunit beta	-3.155	0.053
1.1.1.339 tll; dTDP-6-deoxy-L-talose 4-dehydrogenase (NAD^+)	2.006	0.054
3.4.21.50 E3.4.21.50; lysyl endopeptidase	1.783	0.054
1.6.1.2 pntB; H^+-translocating NAD(P) transhydrogenase subunit beta	-3.155	0.058
2.7.7.59 glnD; [protein-PII] uridylyltransferase	-3.831	0.058

Table S22. KEGG pathways altered (KO level 3) when comparing group resistant to UV irradiation on Day 30 and Day 60 (n = 3) with the group of non-responsive subjects (n = 20) on Day 60.

**Pathway**	**Log2(Fold-change)**	***Q* value**
5. Isomerases; 5.3 Intramolecular oxidoreductases; 5.3.4 Transposing S-S bonds	-1.448	0.016
Cysteine Peptidases; Family C15: pyroglutamyl peptidase I family	-2.668	0.020
ABC Transporters, Prokaryotic Type; Metallic cation, iron-siderophore and vitamin B12 transporters; Iron/zinc/copper transporter [MD:M00318]	-1.274	0.021
2. Transferases; 2.7 Transferring phosphorus-containing groups; 2.7.11 Protein-serine/threonine kinases	-1.168	0.021
3. Hydrolases; 3.4 Acting on peptide bonds (peptidases); 3.4.19 Omega peptidases	-1.122	0.021
Type IV secretion system; Conjugal transfer pilus assembly protein	-1.017	0.021
Major Facilitator Superfamily (MFS); Lipid transporters; Lysophospholipid transporter (LplT) family [TC:2.A.1.42]	-0.930	0.024

Serine/threonine protein kinases: Others; Wnk family	-1.163	0.028

Table S23. Day 15 taxonomic analysis comparing the group resistant to UV irradiation on Day 30 and Day 60 (*n* = 3) with the remaining group subjects responsive only on Day 30 (*n* = 6).

**Taxonomy**	**Log2(Fold-change)**	***Q* value**	**Functional Connotations**
c__Gammaproteobacteria[1]	-5.225	0.060	Associated with endogenous alcohol production suggesting a mechanism for the development of liver damage [29]
s__*Parabacteroides_distasonis*	-1.700	0.061	Probiotic effects [30]
s__*Ruminococcus_bicirculans*	-9.902	0.063	Consistently present in the healthy human gut [31]
s__*Bacteroides_fragilis*	-2.350	0.078	Associated with acute diarrheal, inflammatory bowel disease, and colorectal cancer (CRC) [32]
c__Spirochaetia	-2.676	0.078	Relates to the high fiber intake from ingested plant polysaccharides to produce high levels of short-chain fatty acids and thereby maximizing metabolic energy extraction [33]
g__*Ruminiclostridium*	-4.727	0.078	Consistently present in the healthy human gut [31]
p__Spirochaetes	-2.676	0.088	Association to the etiology of chronic diseases [33]
s__*Phascolarctobacterium_succinatutens*[1]	-1.552	0.094	*Phascolarctobacterium* can produce short-chain fatty acids [34]

[1]Taxonomic hierarchies are designated as c (class), o (order), f (family), g (genus) or s (species).

Table S24. Day 15 comparative enzyme levels when comparing the group resistant to UV irradiation on Day 30 and Day 60 (n = 3) with the remaining group of subjects responsive only on Day 30 (n = 6).

Enzymes	Log2(Fold-change)	P value
2.3.1.117 dapD; 2,3,4,5-tetrahydropyridine-2,6-dicarboxylate N-succinyltransferase	1.131	0.011
3.5.1.28 ampD; *N*-acetyl-anhydromuramoyl-L-alanine amidase	1.516	0.056
1.4.1.21 nadX, ASPDH; aspartate dehydrogenase	-1.092	0.062
3.5.2.6 oxa; beta-lactamase class D	1.117	0.064
2.5.1.144 cysM; *S*-sulfo-L-cysteine synthase (*O*-acetyl-L-serine-dependent)	1.687	0.065
3.5.99.2 tenA; thiaminase (transcriptional activator TenA)	1.082	0.066
1.1.1.44 PGD, gnd, gntZ; 6-phosphogluconate dehydrogenase	-1.354	0.066
2.7.7.19 pcnB; poly(A) polymerase	-1.793	0.066
1.5.5.2 putA; RHH-type transcriptional regulator, proline utilization regulon repressor / proline dehydrogenase / delta 1-pyrroline-5-carboxylate dehydrogenase	-1.398	0.067
3.4.22.8 cloSI; clostripain	-1.405	0.067
1.1.1.343 PGD, gnd, gntZ; 6-phosphogluconate dehydrogenase	-1.354	0.068
2.7.1.16 araB; L-ribulokinase	1.923	0.068
4.2.1.55 croR; 3-hydroxybutyryl-CoA dehydratase	-1.823	0.069
1.2.1.88 putA; RHH-type transcriptional regulator, proline utilization regulon repressor / proline dehydrogenase / delta 1-pyrroline-5-carboxylate dehydrogenase	-1.398	0.069
1.5.1.28 odh; opine dehydrogenase	-1.113	0.070
3.5.4.16 folE2; GTP cyclohydrolase IB	-1.482	0.070
1.16.3.1 bfr; bacterioferritin	1.001	0.070
3.2.1.23 lacZ; beta-galactosidase	-2.789	0.071
3.1.4.53 cpdA; 3',5'-cyclic-AMP phosphodiesterase	-1.406	0.071
2.7.7.7 dnaQ; DNA polymerase III subunit epsilon	-1.792	0.071
3.8.1.8 atzA; atrazine chlorohydrolase	1.354	0.071
1.2.7.10 oorB; oxalate oxidoreductase subunit beta	-4.192	0.071
2.3.1.46 metX; homoserine *O*-acetyltransferase/*O*-succinyltransferase	1.076	0.072
2.3.1.31 metX; homoserine *O*-acetyltransferase/*O*-succinyltransferase	1.076	0.074

2.4.1.291 pglJ; *N*-acetylgalactosamine-*N*,*N*'-diacetylbacillosaminyl-diphospho-undecaprenol 4-alpha-*N*-acetylgalactosaminyltransferase	-1.291	0.075
3.2.1.22 E3.2.1.22B, galA, rafA; alpha-galactosidase	-1.065	0.078
1.1.1.346 dkgB; 2,5-diketo-D-gluconate reductase B	-1.497	0.079
1.4.1.11 kdd; L-erythro-3,5-diaminohexanoate dehydrogenase	1.061	0.080
1.2.4.1 PDHB, pdhB; pyruvate dehydrogenase E1 component beta subunit	1.802	0.080
4.1.1.37 hemE, UROD; uroporphyrinogen decarboxylase	-1.145	0.081
2.4.1.52 tagE; poly(glycerol-phosphate) alpha-glucosyltransferase	-1.245	0.083
1.8.1.8 trxC; thioredoxin 2	-1.626	0.083
1.1.1.403 dthD; D-threitol dehydrogenase (NAD^+)	-5.753	0.083
6.2.1.26 menE; *O*-succinylbenzoic acid---CoA ligase	-1.148	0.083
3.2.1.23 ebgA; evolved beta-galactosidase subunit alpha	-1.031	0.084
3.6.1.65 nudG; (d)CTP diphosphatase	-1.666	0.084
3.2.1.99 abnA; arabinan endo-1,5-alpha-L-arabinosidase	-1.798	0.084
4.2.1.151 mqnA; chorismate dehydratase	1.566	0.085
3.1.3.104 yigB; FMN hydrolase / 5-amino-6-(5-phospho-D-ribitylamino)uracil phosphatase	-1.079	0.086
4.2.2.6 ogl; oligogalacturonide lyase	-1.607	0.088
5.4.3.3 kamD; beta-lysine 5,6-aminomutase alpha subunit	1.070	0.089
3.1.3.102 yigB; FMN hydrolase / 5-amino-6-(5-phospho-D-ribitylamino)uracil phosphatase	-1.079	0.090
1.11.1.9 gpx; glutathione peroxidase	-1.713	0.090
1.1.1.339 tll; dTDP-6-deoxy-L-talose 4-dehydrogenase (NAD+)	2.261	0.091
5.1.3.32 rhaM; L-rhamnose mutarotase	-1.170	0.092
2.5.1.129 ubiX, bsdB, PAD1; flavin prenyltransferase	1.175	0.094
2.7.13.3 comP; two-component system, NarL family, sensor histidine kinase ComP	1.433	0.095
4.2.2.2 pel; pectate lyase	-2.374	0.096
5.1.3.20 gmhD, rfaD; ADP-L-glycero-D-manno-heptose 6-epimerase	-2.096	0.097
2.7.4.6 ndk, NME; nucleoside-diphosphate kinase	-1.278	0.098
3.2.1.18 NEU1; sialidase-1	-1.315	0.099

2.5.1.120 mqnE; aminodeoxyfutalosine synthase	1.333	0.10
5.4.3.3 kamE; beta-lysine 5,6-aminomutase beta subunit	1.223	0.10
1.1.1.17 mtlD; mannitol-1-phosphate 5-dehydrogenase	1.210	0.11
4.1.1.50 speD, AMD1; *S*-adenosylmethionine decarboxylase	-2.551	0.13
3.2.1.83 cgkA; kappa-carrageenase	1.285	0.13

Table S25. Day 15 analysis of KEGG pathways altered (KO level 3) when comparing the group resistant to UV irradiation on Day 30 and Day 60 (*n* = 3) with the remaining group of subjects responsive only on Day 30 (*n* = 6).

**Pathways**	**Log2(Fold-change)**	***Q* value**
Phosphotransferase System (PTS); Enzyme II [TC:4.A]; Galactosamine-specific II component [MD:M00287]	-1.330	0.035
Polysaccharide; Storage polysaccharide	0.521	0.049
ABC Transporters, Prokaryotic Type; Metallic cation, iron-siderophore and vitamin B12 transporters; Zinc transporter [MD:M00242]	0.226	0.050
Major Facilitator Superfamily (MFS); Sugar transporters; Oligosaccharide:H^+ symporter (OHS) family [TC:2.A.1.5]	-2.796	0.053
NarL family; ComP-ComA	1.447	0.053
1. Oxidoreductases; 1.5 Acting on the CH-NH group of donors; 1.5.1 With NAD^+ or $NADP^+$ as acceptor	-0.121	0.056
Phospholipid acyltransferase; LPAAT/LPLAT	0.331	0.056
Aminoacyl-tRNA synthetase; Class II (A)	0.165	0.060
5. Isomerases; 5.4 Intramolecular transferases; 5.4.3 Transferring amino groups	0.667	0.070

Type V secretion system; Autotransporter-1 (AT-1) family	-2.850	0.072

Table S26. Day 30 taxonomic analysis comparing the group resistant to UV irradiation on Day 30 and Day 60 (*n* = 3) with the remaining group of subjects responsive only on Day 30 (*n* = 6).

Taxonomy	Log2(Fold-change)	*Q* value	Functional Connotations
s__*Roseburia_inulinivorans*[1]	1.901	0.050	Contributes to butyrate formation [35]
f__*Acidaminococcaceae*	-2.758	0.064	Regulate the AA utilization and metabolism in small-intestinal bacteria [36]
c__Bacteroidia	0.557	0.068	Production of a pool of volatile fatty acids that are reabsorbed through the large intestine and utilized by the host as an energy source [37]
p__Firmicutes	-0.657	0.072	Related to the protective function of intestinal barrier [38].
o__Acidaminococcales	-2.758	0.085	Regulate the AA (amino acid) utilization and metabolism in small-intestinal bacteria [36]

[1]Taxonomic hierarchies are designated as c (class), o (order), f (family), g (genus) or s (species).

Table S27. Day 30 comparative enzyme levels when comparing the group resistant to UV irradiation on Day 30 and Day 60 (*n* = 3) with the remaining group of subjects responsive only on Day 30 (*n* = 6).

Enzymes	Log2(Fold-change)	*Q* value
3.6.1.65 nudG; (d)CTP diphosphatase	-2.475	0.033
6.2.1.5 sucD; succinyl-CoA synthetase alpha subunit	-1.451	0.033
2.7.1.92 iolC; 5-dehydro-2-deoxygluconokinase	-2.178	0.045
2.1.1.207 trmL, cspR; tRNA (cytidine/uridine-2'-*O*-)-methyltransferase	-1.081	0.045
3.4.21.88 lexA; repressor LexA	-1.185	0.046
2.1.1.295 K18534; MPBQ/MSBQ methyltransferase	4.799	0.047

2.7.7.7 holB; DNA polymerase III subunit delta'	-2.403	0.047
5.4.4.3 glnA; 3-(hydroxyamino)phenol mutase	-1.160	0.047
3.1.4.55 phnP; phosphoribosyl 1,2-cyclic phosphate phosphodiesterase	0.549	0.047
5.3.1.30 iolB; 5-deoxy-glucuronate isomerase	-1.695	0.048
2.7.1.60 nanK; *N*-acylmannosamine kinase	1.699	0.048
2.7.1.107 dgkA, DGK; diacylglycerol kinase (ATP)	-1.473	0.048
2.7.7.75 mogA; molybdopterin adenylyltransferase	-1.047	0.048
3.5.1.87 pydC; beta-ureidopropionase / *N*-carbamoyl-L-amino-acid hydrolase	-3.534	0.048
3.5.4.16 folE2; GTP cyclohydrolase IB	-2.008	0.048
1.2.7.10 oorB; oxalate oxidoreductase subunit beta	-4.109	0.049
1.8.5.4 sqr; sulfide:quinone oxidoreductase	2.089	0.049
3.5.1.6 pydC; beta-ureidopropionase / *N*-carbamoyl-L-amino-acid hydrolase	-3.534	0.050
3.2.1.4 bcsZ; endoglucanase	-2.346	0.051
3.2.1.22 melA; alpha-galactosidase	-1.710	0.051
3.4.22.37 rgpA_B; gingipain R	3.515	0.051
3.6.3.20 ugpC; *sn*-glycerol 3-phosphate transport system ATP-binding protein	-1.390	0.051
4.1.99.22 moaA, CNX2; GTP 3',8-cyclase	-0.888	0.052
5.1.3.20 gmhD, rfaD; ADP-L-glycero-D-manno-heptose 6-epimerase	-2.295	0.055
1.2.7.1 porA; pyruvate ferredoxin oxidoreductase alpha subunit	-1.775	0.055
2.5.1.61 hemC, HMBS; hydroxymethylbilane synthase	-1.007	0.056
6.3.4.5 argG, ASS1; argininosuccinate synthase	-0.328	0.056
5.4.99.19 rsuA; 16S rRNA pseudouridine516 synthase	-1.027	0.058
4.2.1.24 hemB, ALAD; porphobilinogen synthase	-1.353	0.058
1.2.1.70 hemA; glutamyl-tRNA reductase	-1.372	0.058
2.1.1.34 trmH; tRNA (guanosine-2'-*O*-)-methyltransferase	0.670	0.059
3.1.3.10 yihX; glucose-1-phosphatase	0.306	0.060
2.7.7.53 E2.7.7.53; ATP adenylyltransferase	-3.013	0.066

Table S28. Day 30 analysis of KEGG pathways altered (KO level 3) when comparing the group resistant to UV irradiation on Day 30 and Day 60 (n = 3) with the remaining group of subjects responsive only on Day 30 (n = 6).

**Pathways**	**Log2(Fold-change)**	***Q* value**
3. Hydrolases; 3.4 Acting on peptide bonds (peptidases); 3.4.13 Dipeptidases	0.249	0.033
ABC Transporters, Prokaryotic Type; Mineral and organic ion transporters; Iron(III) transporter [MD:M00190]	-1.136	0.034
Heat shock proteins; HSP33	-0.708	0.036
CitB family; CitS-CitT	-1.054	0.037
1. Oxidoreductases; 1.3 Acting on the CH-CH group of donors; 1.3.99 With other acceptors	-0.255	0.039
Serine Peptidases; Family S24: LexA family	-1.170	0.043
2. Transferases; 2.7 Transferring phosphorus-containing groups; 2.7.9 Phosphotransferases with paired acceptors	-1.220	0.044
ABC Transporters, Prokaryotic Type; ABC-2 type and other transporters; Lipoprotein-releasing transporter [MD:M00255]	0.465	0.048
Major Facilitator Superfamily (MFS); Organic acid transporters; Cyanate porter (CP) family [TC:2.A.1.17]	-2.440	0.054
1. Oxidoreductases; 1.4 Acting on the CH-NH2 group of donors; 1.4.3 With oxygen as acceptor	0.286	0.060
ABC Transporters, Prokaryotic Type; Oligosaccharide and polyol transporters; Multiple sugar transporter [MD:M00196]	-0.945	0.068
ABC Transporters, Prokaryotic Type; Oligosaccharide and polyol transporters; Putative *sn*-Glycerol 3-phosphate transporter [MD:M00198]	-1.339	0.096

Supplementary File S6. Metabolomic analyses

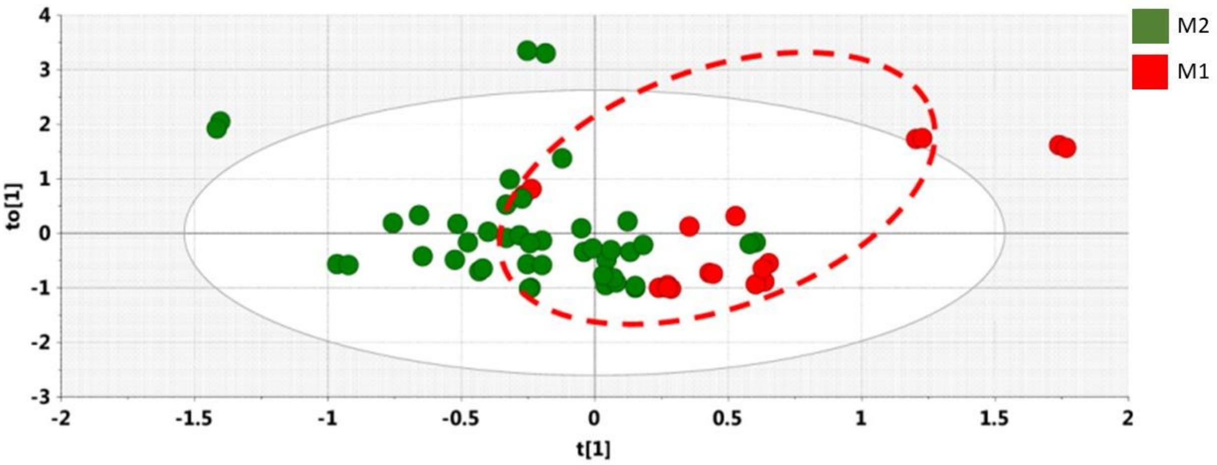

Figure S3. OPLS-DA Scores plot for Day 15 plasmas vs. Day 30 plasmas showing UV-resistant subjects (M1; *n* = 9; red) and non-resistant subjects (M2; *n* = 20; green). Data presented as duplicate analyses. No statistically significant metabolites were discerned from this plasma study.

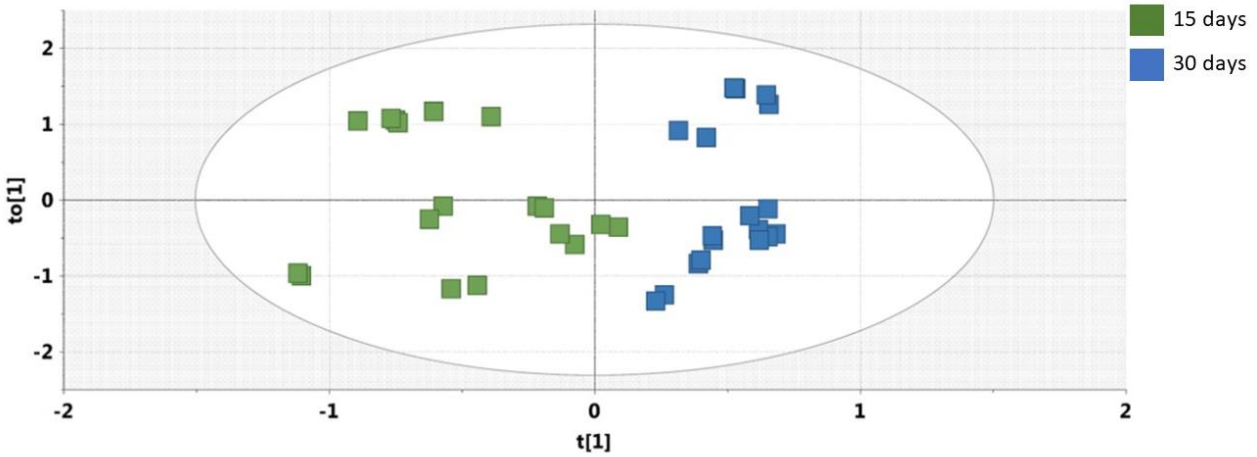

Figure S4. OPLS-DA Scores plot for Day 15 plasmas (green) vs. Day 30 plasmas (blue) for the UV-resistant subjects (*n* = 9). Data presented as duplicate analyses.

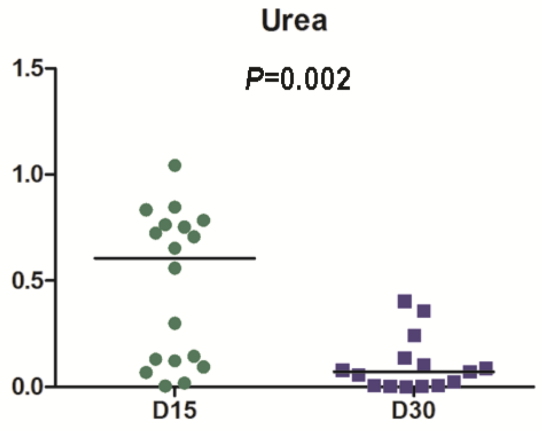

Figure S5. Univariate analysis (Mann-Whitney) on plasma metabolites for UV resistant subjects (n = 9) at Day 15 (D15; following a restricted diet) vs. Day 30 (D30; following two weeks of grape consumption). Data presented as duplicate analyses. P-value is Bonferroni corrected.
